# Structural and Functional Characterization of a Novel Scorpion Toxin that Inhibits Na_V_1.8 via Interactions With the DI Voltage Sensor and DII Pore Module

**DOI:** 10.3389/fphar.2022.846992

**Published:** 2022-05-19

**Authors:** Kiran George, Diego Lopez-Mateos, Tarek Mohamed Abd El-Aziz, Yucheng Xiao, Jake Kline, Hong Bao, Syed Raza, James D. Stockand, Theodore R. Cummins, Luca Fornelli, Matthew P. Rowe, Vladimir Yarov-Yarovoy, Ashlee H. Rowe

**Affiliations:** ^1^ Department of Biology, University of Oklahoma, Norman, OK, United States; ^2^ Department of Physiology and Membrane Biology, University of California, Davis, Davis, CA, United States; ^3^ Biophysics Graduate Group, University of California, Davis, Davis, CA, United States; ^4^ Department of Cellular and Integrative Physiology, University of Texas Health Science Center San Antonio, San Antonio, TX, United States; ^5^ Zoology Department, Faculty of Science, Minia University, El-Minia, Egypt; ^6^ Amsaal Venom Farm L.L.C., Abu Dhabi, United Arab Emirates; ^7^ Department of Biology, School of Science, Indiana University-Purdue University Indianapolis, Indianapolis, IN, United States; ^8^ Department of Anesthesiology and Pain Medicine, University of California, Davis, Davis, CA, United States; ^9^ Graduate Program in Cellular and Behavioral Neurobiology, University of Oklahoma, Norman, OK, United States

**Keywords:** Nav1.8, voltage-gated sodium channel, AZ bark scorpion, grasshopper mice, NaTx36, slow inactivation, venom, neurotoxin

## Abstract

Voltage-gated sodium channel Na_V_1.8 regulates transmission of pain signals to the brain. While Na_V_1.8 has the potential to serve as a drug target, the molecular mechanisms that shape Na_V_1.8 gating are not completely understood, particularly mechanisms that couple activation to inactivation. Interactions between toxin producing animals and their predators provide a novel approach for investigating Na_V_ structure-function relationships. Arizona bark scorpions produce Na^+^ channel toxins that initiate pain signaling. However, in predatory grasshopper mice, toxins inhibit Na_V_1.8 currents and block pain signals. A screen of synthetic peptide toxins predicted from bark scorpion venom showed that peptide NaTx36 inhibited Na^+^ current recorded from a recombinant grasshopper mouse Na_V_1.8 channel (OtNa_V_1.8). Toxin NaTx36 hyperpolarized OtNa_V_1.8 activation, steady-state fast inactivation, and slow inactivation. Mutagenesis revealed that the first gating charge in the domain I (DI) S4 voltage sensor and an acidic amino acid (E) in the DII SS2 – S6 pore loop are critical for the inhibitory effects of NaTx36. Computational modeling showed that a DI S1 – S2 asparagine (N) stabilizes the NaTx36 – OtNa_V_1.8 complex while residues in the DI S3 – S4 linker and S4 voltage sensor form electrostatic interactions that allow a toxin glutamine (Q) to contact the first S4 gating charge. Surprisingly, the models predicted that NaTx36 contacts amino acids in the DII S5 – SS1 pore loop instead of the SS2 – S6 loop; the DII SS2 – S6 loop motif (QVSE) alters the conformation of the DII S5 – SS1 pore loop, enhancing allosteric interactions between toxin and the DII S5 – SS1 pore loop. Few toxins have been identified that modify Na_V_1.8 gating. Moreover, few toxins have been described that modify sodium channel gating via the DI S4 voltage sensor. Thus, NaTx36 and OtNa_V_1.8 provide tools for investigating the structure-activity relationship between channel activation and inactivation gating, and the connection to alternative pain phenotypes.

## Introduction

**FIGURE 1:**
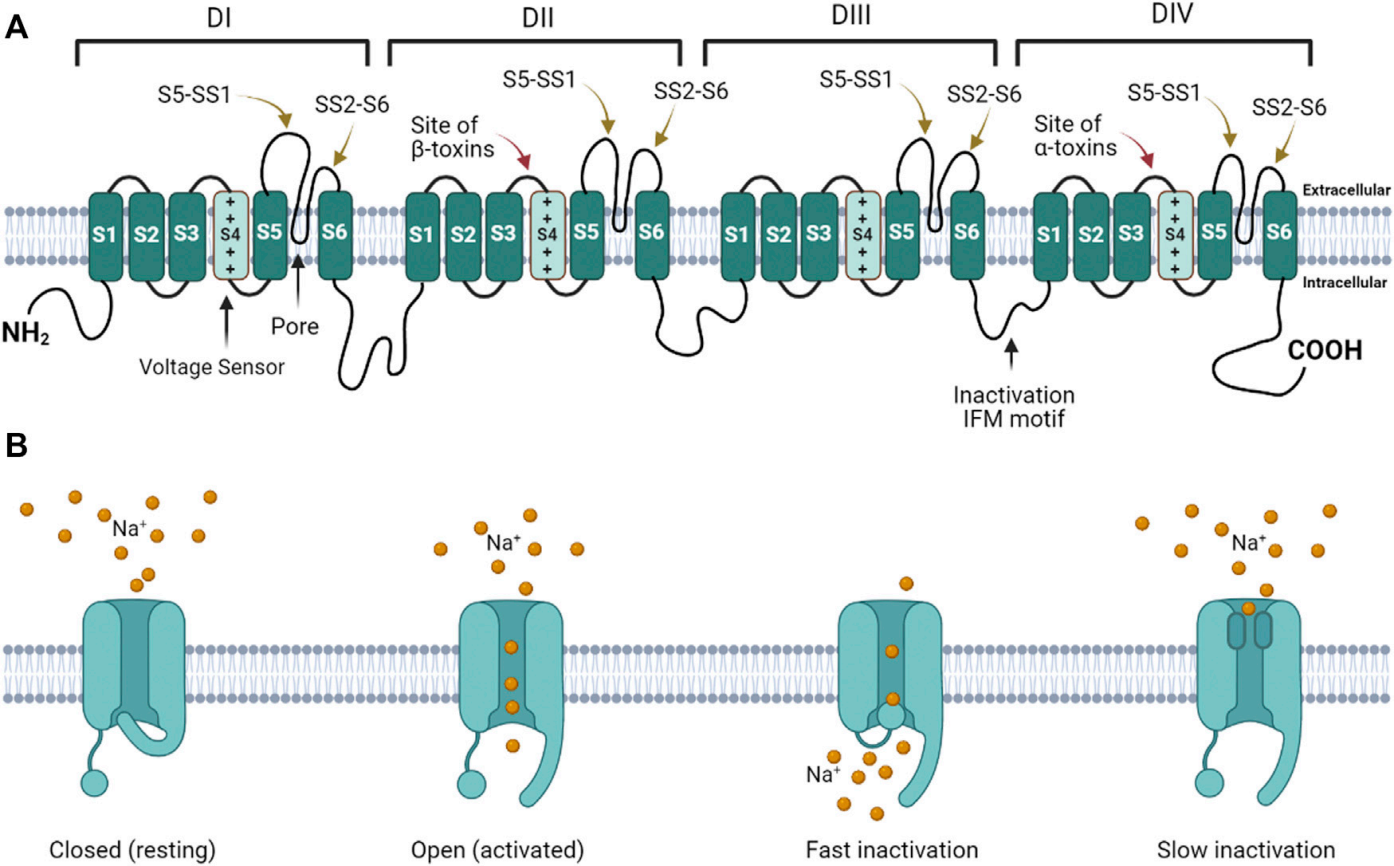
Voltage gated sodium channel structure and function. **(A)** The channel consists of four homologous domains (DI – DIV). Each domain consists of six transmembrane helices (S1 – S6). The fourth helix (S4) in each domain contains basic amino acids that act as voltage-sensor gating charges. The re-entrant loops between S5 and S6 in each domain form the pore (black arrow points to pore in DI). The extracellular loops connecting S5 to the pore (S5 – SS1) and the pore to S6 (SS2 – S6) form the mouth of the pore. The intracellular loop connecting DIII to DIV forms the fast inactivation hinged lid. The isoleucine-phenylalanine-methionine (IFM) motif is critical for fast inactivation gating. The DII and DIV S3 – S4 linkers form the primary scorpion β and α toxin binding sites, respectively. **(B)** When the channel is in the resting state, the activation gate is closed. When the channel is activated, the gate opens. During activation the inactivation gate (hinged lid) is disengaged. During fast inactivation, the hinged lid occludes the intracellular side of the pore. When the channel transitions into the slow inactivated state, the pore loops change conformation to obstruct the pore.

Voltage gated sodium ion channels (Na_V_) are transmembrane protein pores that generate the action potentials underlying neuronal signaling and muscle contraction (Catterall, 1980; Catterall, 1992; Catterall, 2000; Catterall et al., 2005; Ahern et al., 2016). Mammals express nine genes that encode Na_V_ isoforms expressed in different tissues and at different developmental time points (Ahern et al., 2016). Na_V_ generate action potentials by regulating the flux of Na^+^ ions across excitable cell membranes (Catterall, 1992; Catterall, 2000). These channels are activated by voltage (initiating Na^+^ influx), and inactivated by terminating Na^+^ flux (Catterall, 2014). Na_V_ have four domains, each consisting of six helices (S1 – S6) (Figure 1A). In each domain, the transmembrane re-entrant loop between S5 and S6 form the ion-permeating pore and Na^+^ selectivity filter, while the extracellular loops that link S5 to the pore (S5 – SS1) and the pore to S6 (SS2 – S6) form the mouth of the pore. Positively charged amino acids in the S4 segment of each domain function as voltage sensor gating charges (GC). The intracellular loop that links DIII and DIV contains an isoleucine-phenylalanine-methionine (IFM) motif that forms the fast inactivation mechanism. The extracellular loops linking S3 to S4 in DII and DIV serve as binding sites for scorpion β and α toxins, respectively. At resting membrane potential, the channel is closed (Figure 1B). Depolarization of the cell membrane alters the electrostatic forces that move S4 helices outward, opening the channel (Catterall, 2000; Catterall, 2014). While the movement of the S4 helices in DI – DIII are associated with channel activation (opening), the movement of the S4 helix in DIV initiates the fast inactivation mechanism (Armstrong, 2006). During fast inactivation, the DIII – DIV loop acts as an inactivation particle to occlude the pore. Slow inactivation is a second form of inactivation that involves a rearrangment of the segments that line the pore (Vilin and Ruben, 2001; Silva, 2014; Chatterjee et al., 2018).

Three Na_V_ (Nav1.7, 1.8, 1.9) are expressed in peripheral nociceptive neurons where they contribute to membrane excitability (Vilin and Ruben, 2001; Blair and Bean, 2002; Blair and Bean, 2003; Sokolov et al., 2008). Na_V_1.8 generates the majority of Na^+^ current underlying the upstroke of action potentials in small-diameter nociceptive neurons, and thus, is a key ion channel governing the excitability of sensory neurons and transmission of pain signals (Cummins and Waxman, 1997; Renganathan et al., 2001; Blair and Bean, 2002; Blair and Bean, 2003; Cummins et al., 2007; Dib-Hajj et al., 2010). Numerous studies have implicated Na_V_1.8 currents in mechanical, cold, neuropathic, and inflammatory pain – highlighting the potential for Na_V_1.8 to serve as a target for drug therapy (Cummins et al., 2007; Basbaum et al., 2009; Cavanaugh et al., 2009; Le Pichon and Chesler, 2014; Barbosa et al., 2016; Peirs and Seal, 2016). While progress has been made elucidating the role of Na_V_1.8 in regulating the excitability of nociceptive neurons and transmission of pain signals, the molecular mechanisms that govern Na_V_1.8 gating are not completely understood. Animal-derived venoms and peptide toxins are useful tools for investigating Na_V_ structure-function relationships. For example, scorpion toxins were used to examine the structure and function of voltage sensors (Na_V_1.2) and the fast inactivation mechanism (Na_V_1.7) (Cestèle et al., 1998; Bosmans and Tytgat, 2007; Bosmans and Swartz, 2010; Zhang et al., 2011; Clairfeuille et al., 2019). However, until recently, few animal toxins had been identified that modify Na_V_1.8 gating (Ye et al., 2015), prompting efforts to construct chimeras of Na_V_1.8 that could bind peptide toxins by exchanging extracellular loops on Na_V_1.8 with corresponding toxin binding sites from Na_V_1.2 (Gilchrist and Bosmans, 2018). More recently, additional peptide toxins have been discovered that modify Na_V_1.8 gating, providing insight into structure-activity relationships between voltage sensors and gating mechanisms (Zhang et al., 2019; Deuis et al., 2021; Finol-Urdaneta et al., 2022).

Toxin-producing animals and their predators provide an alternative approach for using toxin – Na_V_ interactions to examine the relationship between voltage sensors and gating processes. Arizona (AZ) bark scorpions (Centruroides sculpturatus) produce venom that is painful as well as potentially lethal (Curry et al., 1983; Boyer et al., 2009). The venom is a cocktail of peptide toxins that bind Na^+^ and K^+^ channels in nerve and muscle tissue (Couraud et al., 1984; Simard et al., 1992; Possani et al., 1999; Possani et al., 2000; Corona et al., 2001; Rodríguez de la Vega and Possani, 2004; Rodríguez de la Vega and Possani, 2005). The peptides do not cause pain by damaging tissue; they activate Na_V_1.7, hyperexciting nociceptive neurons (Vandendriessche et al., 2010; Rowe et al., 2011; Rowe et al., 2013). Anecdotal reports describe the venom as producing the sensation of burning pain, coupled with hypersensitivity to touch and pressure. Thus, bark scorpion venom provides a source of novel biochemicals for probing ion channels that regulate nociceptive neuron excitability and pain-related behavior. Southern grasshopper mice (Onychomys torridus) prey on bark scorpions. Compared to house mice, grasshopper mice show little response when either stung by scorpions or injected with venom (Rowe and Rowe, 2006; Rowe and Rowe, 2008; Rowe et al., 2013). Electrophysiological analyses showed that bark scorpion venom inhibited Na_V_1.8 currents and blocked the propagation of action potentials in dissociated, small-diameter dorsal root ganglia (DRG) neurons from grasshopper mice (Rowe et al., 2013). In contrast, bark scorpion venom had no effect on house mice Na_V_1.8 currents. Instead of blocking action potentials in house mice DRG neurons, the venom increased the propagation of action potentials. Moreover, pre-injecting grasshopper mice with venom decreased their pain-related behavior in response to formalin. Pre-injecting house mice with venom only increased their pain related behavior in response to formalin. Collectively, the findings demonstrate that grasshopper mice have evolved resistance to painful venom. Our hypothesis is that grasshopper mice have amino acid variants in Na_V_1.8 that enable the channel to bind peptide toxins which inhibit channel activity and block pain signals. Thus, bark scorpions and grasshopper mice provide a novel system for investigating Na_V_1.8 amino acid variants that alter the structure-activity relationship between the channel and venom peptides, and for determining the biophysical mechanisms that decrease nociceptive-neuron excitability and pain-related behavior in a rodent model.

We previously isolated a venom subfraction (F11-E) from the AZ bark scorpion and showed that it inhibited tetrodotoxin resistant (TTX-R) Na^+^ current recorded from the grasshopper mouse recombinant OtNa_V_1.8 channel (Mohamed Abd El-Aziz et al., 2021). Using liquid chromatography mass spectrometry (LC MS), we showed that subfraction F11-E contained four peptides each having the mass and primary structure characteristic of scorpion sodium channel toxins (NaTx) (Mohamed Abd El-Aziz et al., 2021). The AZ bark scorpion venom gland transcriptome (NCBI GenBank accession number PRJNA340270) was used to confirm the primary structure of the novel peptides (NaTx4, NaTx13, NaTx22, NaTx36) (Mohamed Abd El-Aziz et al., 2021). Our goal is to identify peptides that inhibit OtNa_V_1.8 and to functionally characterize the inhibitory activity. To this end, the peptides were chemically synthesized by SB-PEPTIDE (SmartBioscience SAS, France) and then tested on OtNa_V_1.8. Here, we report that NaTx36 inhibits OtNa_V_1.8 TTX-R Na^+^ current in a concentration and voltage dependent manner, recapitulating the inhibitory effects of AZ bark scorpion venom on OtNa_V_1.8. Electrophysiological analyses show that NaTx36 modulates OtNa_V_1.8 activation and inactivation gating, while site-directed mutagenesis and computational modeling suggest that amino acids in the DI voltage sensor and the DII pore module are critical for channel inhibition. These results are significant because few toxins have been identified that target Na_V_1.8 (Ye et al., 2015; Zhang et al., 2019; Deuis et al., 2021). Moreover, few toxins have been described that modify Na_V_ gating mechanisms via interaction with the DI voltage sensor (Xiao et al., 2014; Clairfeuille et al., 2019). The interactions between NaTx36 and OtNa_V_1.8 provide a toolkit for investigating the structure-activity relationship between channel activation and inactivation gating mechanisms.

## Results

### NaTx36 Inhibits Recombinant Grasshopper Mouse OtNa_V_1.8 Na^+^ Current

We used whole-cell patch clamp electrophysiology to measure the effects of synthetic peptide toxins on TTX-R Na^+^ current recorded from OtNa_V_1.8 expressed in ND7/23 cells. Custom peptide toxins were synthesized by SB-PEPTIDE (SmartBioscience SAS, France) using the amino acid sequence of the primary structures previously determined for NaTx36, NaTx22, NaTx13, NaTx4 (Mohamed Abd El-Aziz et al., 2021). SB-PEPTIDE purified the final peptides using High Performance Liquid Chromatography (HPLC), confirmed the mass using Liquid Chromatography Mass Spectrometry (LC MS), and quantified the peptides using OD280. The purity, intact mass and amino acid sequence of each peptide was validated in this study using LC MS, and bottom-up MS/MS, respectively (Supplementary Figures S1 –S8).

**FIGURE 2:**
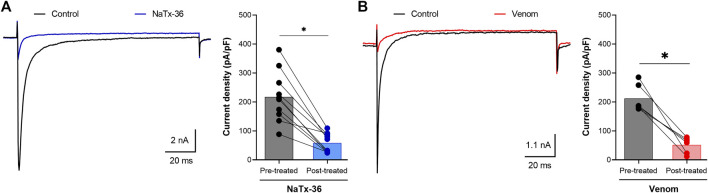
Effects of NaTx36 on grasshopper mouse recombinant OtNa_V_1.8 Na^+^ current density. Na^+^ currents were elicited by a 100-ms depolarization to +20 mV from a holding potential of −80 mV. Tetrodotoxin (TTX, 500 nM) was added to the bath solution to block endogenous TTX sensitive Na^+^ currents. Representative Na^+^ current traces and current density (pA/pF) recorded from whole-cell voltage-clamped ND7/23 cells transfected with OtNa_V_1.8. Na^+^ currents were recorded before and after application of NaTx36 and AZ bark scorpion venom. **(A)** NaTx36 (blue symbols, 10 μg/ml, *n* = 9 cells), and **(B)** venom (red symbols, 1.0 μg/ml, *n* = 6 cells) inhibited tetrodotoxin resistant (TTX-R) Na^+^ currents. Filled circles represent current density values for individually recorded cells. Histograms represent mean current density reported as picoamps divided by picofarads (pA/pF, * *p* < 0.05).

A sample of each peptide toxin was separately diluted in bath solution to the desired final concentration. Na^+^ currents were elicited by a 100-millisecond depolarization to +20 mV from a holding potential of −80 mV before and after application of toxins. Toxin NaTx36 significantly decreased OtNa_V_1.8 Na^+^ current density from 217.5 ± 27.83 pA/pF to 57.82 ± 10.47 pA/pF (10 μg/ml, *n* = 9 cells, *p* < 0.05) (Figure 2A). The inhibitory effect of NaTx36 was characteristic of the effect of AZ bark scorpion venom on OtNa_V_1.8, with the exception that a lower concentration of venom is required to inhibit OtNa_V_1.8. Venom decreased current density from 212.1 ± 19.18 pA/pF to 51.56 ± 11.25 pA/pF (1.0 μg/ml, *n* = 6 cells, *p* < 0.05) (Figure 2B).

Synthetic peptide toxins NaTx4, NaTx22, and NaTx13 were also tested on OtNa_V_1.8 using the protocol described above (Supplementary Figure S9A). NaTx4 (pre-treated: 255.7 ± 31.05 pA/pF, post-treated: 251.3 ± 29.45 pA/pF, *n* = 7 cells), NaTx22 (pre-treated: 220.5 ± 32.46 pA/pF, post-treated: 196.4 ± 29.45 pA/pF, *n* = 8 cells), and NaTx13 (pre-treated: 240.3 ± 31.01 pA/pF, post-treated: 223.3 ± 25.77 pA/pF, *n* = 9 cells) had no effect on OtNa_V_1.8 current density (all toxins tested at 10 μg/ml) (Supplementary Figures S9B–D). These results demonstrate that peptides NaTx4, NaTx22 and NaTx13 are not biologically active against OtNav1.8. Given that the peptides were chemically synthesized (SB-PEPTIDE, SmartBioscience SAS, France), it is possible that the peptides were not bio-active because they did not fold properly.

Our previous work demonstrated that AZ bark scorpion venom had no effect on house mouse Na_V_1.8 (Rowe et al., 2013). An alignment of grasshopper mouse Na_V_1.8 with house mouse sequence revealed variation in the position of an acidic residue (E) within the DII SS2 – S6 loop. In grasshopper mice, the position of E (859QVSE862) is shifted by three amino acids compared to house mice (859EVSQ862). Site-directed mutagenesis showed that the position of the E is critical for the inhibitory effects of AZ bark scorpion venom (Rowe et al., 2013). Thus, we hypothesized that grasshopper mice had evolved amino acid substitutions that enabled their Na_V_1.8 to bind venom peptides and block channel activity. Because human Na_V_1.8 is like the house mouse channel, expressing the E at position 859 (Rowe et al., 2013) (see also Figure 12B in this study), we predicted that NaTx36 would have no effect on human Na_V_1.8 (hNa_V_1.8). To determine whether NaTx36 inhibits human Na_V_1.8, we tested the peptide on a recombinant hNa_V_1.8 channel expressed in ND7/23 cells. Na^+^ currents were elicited by a 50-ms depolarizing pulse to +10 mV before and after application of NaTx36 (Supplementary Figure S10A). To determine the effect of NaTx36 on the hNa_V_1.8 current-voltage relationship, Na^+^ currents were induced by 50-ms depolarizing steps to various potentials ranging from −80 to +40 mV in 5-mV increments (Supplementary Figure S10B). Neither 100 nM nor 1.0 µM NaTx36 inhibited hNav1.8.

### NaTx36 Inhibits OtNa_V_1.8 Activity in a Concentration-Dependent Manner

**FIGURE 3:**
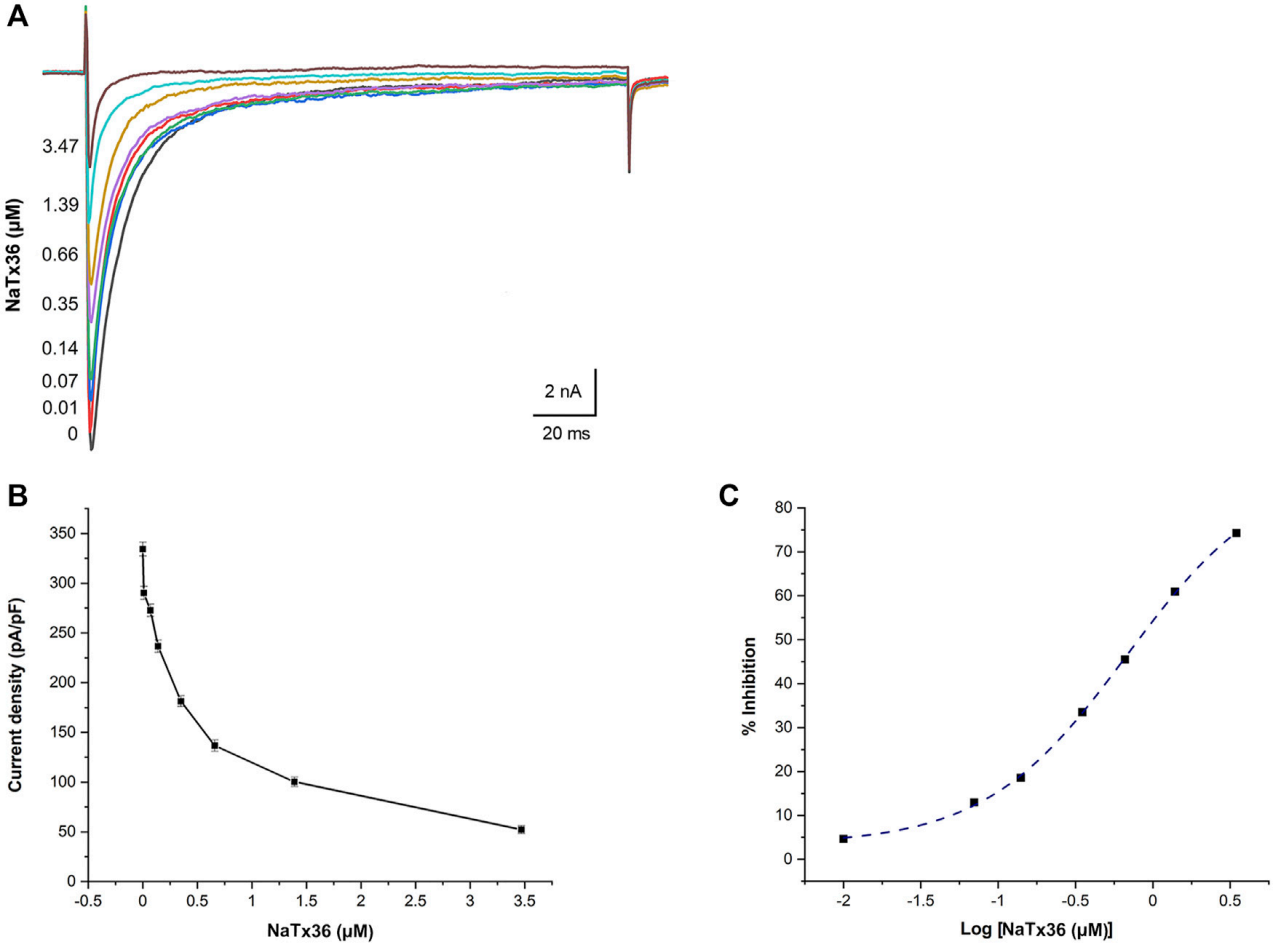
NaTx36 inhibits OtNa_V_1.8 in a concentration dependent manner. **(A)** TTX-R Na^+^ current traces and **(B)** current density (pA/pF) recorded from ND7/23 cells expressing OtNav1.8 before and after application of NaTx36. Na^+^ currents were elicited by a 100-ms depolarization to +20 mV from a holding potential of −80 mV before and after application of NaTx36. Tetrodotoxin (TTX, 500 nM) was added to the bath solution to block endogenous TTX sensitive Na^+^ currents. Currents decrease in response to samples of NaTx36 ranging from 0.01 to 3.47 µM (*n* = 5 – 6 cells per concentration). **(C)** Na^+^ current values in response to NaTx36 were fit to a Dose-response equation with variable Hill slope to estimate the half-maximum inhibitory concentration (IC_50_ = 0.68 ± 0.05 µM).

We previously showed that AZ bark scorpion venom inhibits OtNa_V_1.8 TTX-R Na^+^ current in a concentration-dependent manner (Rowe et al., 2013). To evaluate the dose response effect of NaTx36, we applied a range of toxin concentrations to OtNa_V_1.8 expressed in ND7/23 cells. Na^+^ currents were elicited by a 100-millisecond depolarization to +20 mV from a holding potential of −80 mV before and after application of NaTx36. Similar to the inhibitory effect of venom, increasing concentrations of NaTx36 decreased OtNa_V_1.8 current density (0 μM = 334.33 ± 7.02 pA/pF, 0.1 μM = 290.16 ± 5.53 pA/pF, 0.07 μM = 272.66 ± 4.16 pA/pF, 0.14 μM = 236.66 ± 7.26 pA/pF, 0.35 μM = 181.33 ± 5.38 pA/pF, o.66 μM = 136.66 ± 6.77 pA/pF, 1.39 μM = 100.33 ± 4.83 pA/pF, 3.47 μM = 52.33 ± 4.63 pA/pF, *n* = 5 – 6 cells per concentration) (Figures 3A,B). The half-maximum inhibitory concentration (IC_50_) of NaTx36 = 0.68 ± 0.05 µM (Figure 3C).

### Hyperpolarizing Holding Potentials Reduce NaTx36 Inhibitory Effects on OtNa_V_1.8

**FIGURE 4:**
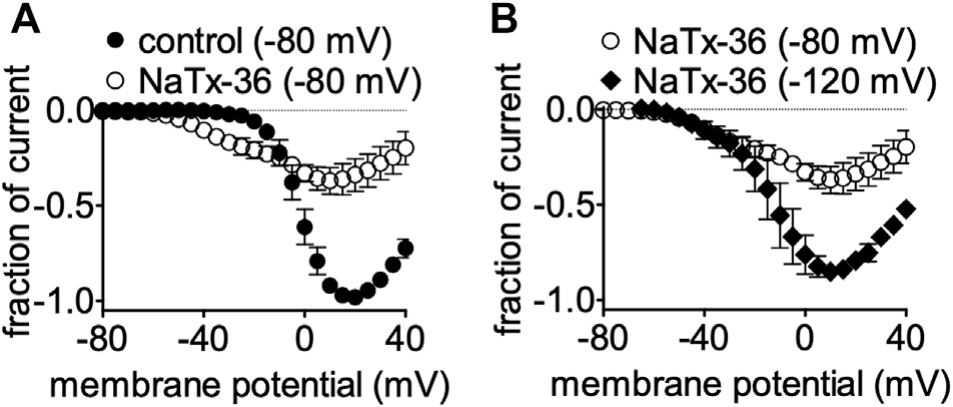
Effects of NaTx36 on OtNa_V_1.8 current-voltage relationship (I-V curves). Cells were pretreated with 500 nM TTX to block TTX-sensitive sodium channels, and currents were elicited by 50-ms depolarizing steps to various voltages ranging from −80 to +40 mV in 5-mV increments. All Na^+^ currents were normalized to the maximum amplitude of control peak current. **(A)** Representative I-V curves before and after 100 nM NaTx36. When the holding potential was −80 mV, NaTx36 could inhibit TTX-R Na^+^ current recorded from ND7/23 cells transfected with recombinant OtNav1.8 (control, *n* = 5; NaTx36, *n* = 4). NaTx36 also enhanced activation by hyperpolarizing the membrane potential at which channels begin opening. **(B)** When the holding potential was changed to −120 mV, inhibition of OtNa_V_1.8 by 100 nM NaTx36 was substantially reduced (−80 mV, *n* = 4; −120 mV, *n* = 3).

We previously showed that the inhibitory effect of venom is voltage-dependent (Mohamed Abd El-Aziz et al., 2021). To determine if the inhibitory effect of NaTx36 on OtNa_V_1.8 is also voltage dependent, we tested NaTx36 on OtNa_V_1.8 at two different holding potentials. Currents were induced by 50-ms depolarizing steps to various potentials ranging from −80 to +40 mV in 5-mV increments. All currents induced before and after application of NaTx36 were normalized to the maximum amplitude of control peak current. At +20 mV, NaTx36 (100 nM) reduced Na^+^ current amplitude by 66.0 ± 0.9% (control *n* = 5 cells, NaTx36 *n* = 4 cells) (Figure 4A). However, when cells were hyperpolarized to a holding potential of −120 mV, the inhibitory effects of NaTx36 (100 nM) were reduced. At +20 mV, the toxin inhibition was reduced from 66.0 ± 0.9% to 20.8 ± 0.8% (−80 mV, *n* = 4 cells; −120 mV, *n* = 3 cells) (Figure 4B). In addition, the results show that NaTx36 enhances OtNa_V_1.8 activation at more hyperpolarized membrane potentials (Figures 4A,B). This suggests that NaTx36 acts as a typical scorpion beta toxin by opening channels at more hyperpolarized membrane potentials (Qu et al., 1998; Cestèle and Catterall, 2000; Mantegazza and Cestèle, 2005).

### NaTx36 Shifts the Voltage Dependence of OtNa_V_1.8 Steady-State Fast and Slow Inactivation

**FIGURE 5:**
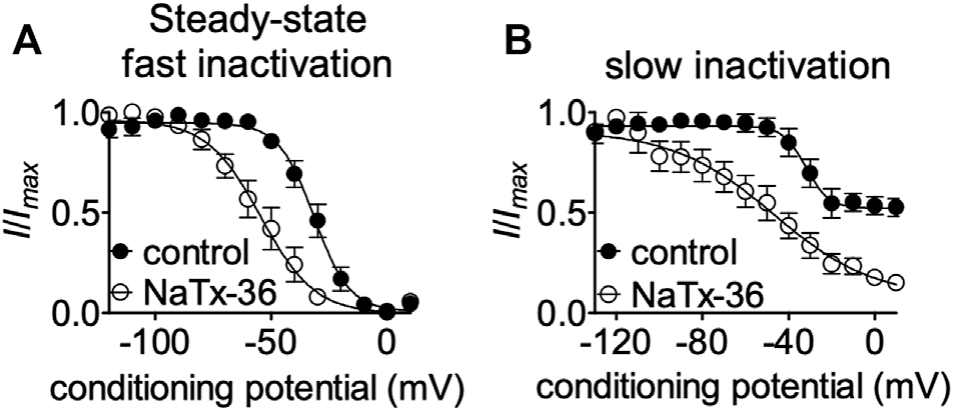
Effects of NaTx36 on OtNav1.8 steady-state fast and slow inactivation. **(A,B)** Representative steady-state fast inactivation and slow inactivation curves before and after 100 nM NaTx36 application to ND7/23 cells transfected with recombinant OtNav1.8. Cells were pretreated with 500 nM TTX to block TTX-sensitive sodium channels. All Na^+^ currents were normalized to the maximum amplitude of control peak current. **(A)** Using a double-pulse protocol, Na^+^ currents were induced by a 20-ms depolarizing potential of +30 mV following a 500-ms prepulse at voltages ranging from −120 to +10 mV. Currents were plotted as a fraction of the maximum peak current. NaTx36 shifted the voltage dependence of steady-state fast inactivation to hyperpolarized membrane potentials (control, *n* = 5 cells; NaTx36, *n* = 3 cells). **(B)** Using a standard stimulus protocol, slow inactivation was induced with 5 s pre-pulses ranging from −130 to +10 mV, followed by 10 ms pulses to −80 mV to allow recovery from fast inactivation. A 20 ms test pulse to +30 mV was then used to determine the fraction of current available. Currents were plotted as a fraction of the maximum peak current. NaTx36 shifted the voltage dependence of slow inactivation to hyperpolarized membrane potentials (control, *n* = 5 cells; NaTx36, *n* = 4 cells).

To gain insight into mechanisms underlying NaTx36 inhibition of OtNa_V_1.8, we determined the effects of the toxin on steady-state fast and slow inactivation. ND7/23 cells were transfected with OtNa_V_1.8 and then pretreated with 500 nM TTX to block TTX-sensitive sodium channels. The voltage dependence of steady-state fast inactivation was measured with a double-pulse protocol where Na^+^ currents were induced by a 20-ms depolarizing potential of +30 mV following a 500-ms prepulse at voltages ranging from −120 to +10 mV. Currents were plotted as a fraction of the maximum peak current. NaTx36 (100 nM) shifted the voltage dependence of steady-state fast inactivation to hyperpolarized membrane potentials (control, *n* = 5 cells; NaTx36, *n* = 3 cells) (Figure 5A).

Next, we asked whether NaTx36 alters the steady-state slow inactivation curve. Using a standard stimulus protocol, slow inactivation was induced with 5 s pre-pulses ranging from −130 to +10 mV, followed by 10 ms pulses to −80 mV to allow recovery from fast inactivation. A 20 ms test pulse to +30 mV was then used to determine the fraction of current available. Currents were plotted as a fraction of the maximum peak current. NaTx36 (100 nM) shifted the voltage dependence of slow inactivation to hyperpolarized membrane potentials (control, *n* = 5 cells; NaTx36, *n* = 4 cells) (Figure 5B).

### Site-Directed Mutagenesis: Residues in the DI S4 Voltage Sensor and DII SS2 – S6 Pore Loops Are Critical for the Inhibitory Effects of Venom and NaTx36

**FIGURE 6:**
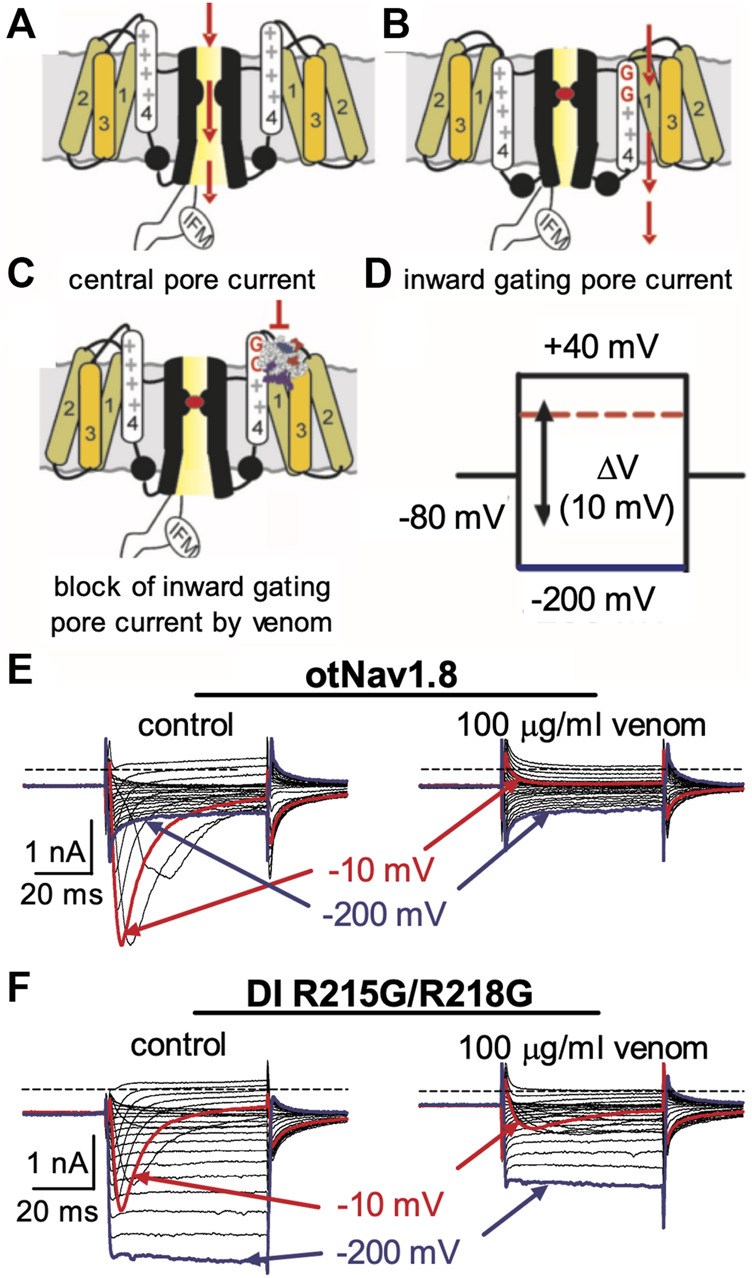
Effects of AZ bark scorpion venom on DI inward gating pore currents. Gating pore currents were generated by glycine mutation of the first and second gating charges (two outermost arginine residues) on OtNa_V_1.8 DI S4 (R215G/R218G). **(A–C)** Schematic diagram depicting channel central pore current **(A)**, generation of inward gating-pore current **(B)**, and block of inward gating pore current by venom **(C)**. **(D)** A typical protocol was used to elicit inward gating pore current. Cells held at −80 mV were stimulated by 50-ms hyperpolarizing steps to various potentials that ranged from –200 to +40 mV in 10-mV increments. **(E)** 100 μg/ml venom completely inhibited central pore current of wild type OtNa_V_1.8 channels but failed to affect background leak currents. **(F)** 100 μg/ml venom only partially inhibited central pore currents recorded from OtNa_V_1.8 mutant channels, suggesting that the double mutation (R215G/R218G) on DI S4 could reduce venom binding affinity towards OtNa_V_1.8 channels. Venom also depressed inward gating pore currents through the DI voltage sensor domain (VSD). Blue and red traces represent typical leak current (gating pore current) and central pore current at −200 and −10 mV, respectively.

**FIGURE 7:**
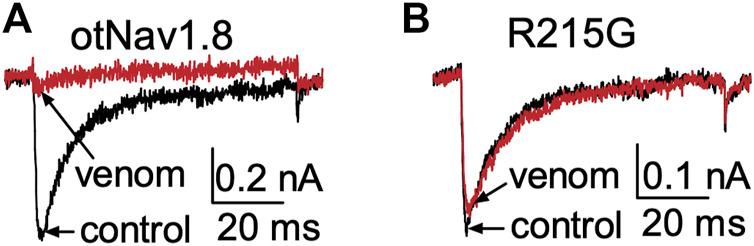
Effects of AZ bark scorpion venom on Na^+^ currents recorded from a DI S4 voltage sensor mutant. **(A)** Venom inhibits wildtype OtNav1.8 Na+ currents. **(B)** The first GC arginine (R215) in the DI S4 voltage sensor was exchanged for a glycine (R215G). TTX (500 nM) was added to the bath solution to block TTX-sensitive currents. Na^+^ currents were elicited by a 50 ms depolarization to +20 mV from a holding potential of −80 mV before and after application of venom (10 μg/ml). Venom inhibited wildtype OtNav1.8, but not the DI S4 voltage sensor mutant (R215G) channels expressed in ND7/23 cells (*n* = 3).

We previously showed that AZ bark scorpion venom inhibited grasshopper mouse Na_V_1.8 TTX-R Na^+^ current while having no effect on house mouse Na_V_1.8 (Rowe et al., 2013). This suggested that grasshopper mice had evolved amino acid substitutions in their Na_V_1.8 that enabled venom peptides to bind the channel and block Na^+^ current. We sequenced the gene (Scn10a) encoding grasshopper mice Na_V_1.8 and found a four amino acid motif (859QVSE862) in the DII SS2 – S6 pore loop that differed from house mice (859EVSQ862) by altering the position of an acidic residue (Rowe et al., 2013). We used site-directed mutagenesis to mutate the OtNa_V_1.8 DII SS2 – S6 loop to exchange the glutamine for the glutamic acid at position 859 (Q859E) and the glutamic acid for the glutamine at position 862 (E862Q), and demonstrated that the 859QVSE862 motif is critical for the inhibitory effects of bark scorpion venom (Rowe et al., 2013). Insertion of the glutamic acid at position 862 into the house mouse recombinant Na_V_1.8 (mNa_V_1.8) inhibited approximately 30% of the TTX-R Na^+^ current, demonstrating that the E862 is necessary but not sufficient for the inhibitory effects of venom (Rowe et al., 2013). This suggests that other amino acids in the grasshopper mouse OtNa_V_1.8 channel contribute to the docking and/or inhibitory activity of venom peptides. Given that the inhibitory effects of the venom are voltage dependent, we reasoned that a voltage sensor might be involved in the mechanism. To test this, we mutated the first and second GC in the OtNa_V_1.8 DI S4 (R215G/R218G) and DII S4 (R756G/R759G) voltage sensors and measured the effect of venom on gating pore currents. Gating pore currents can serve as tools to examine the specificity with which toxins modulate Na_V_ (Figures 6A–C) (Xiao et al., 2014). Cells held at −80 mV were stimulated by 50-ms hyperpolarizing steps to various potentials that ranged from –200 to +40 mV in 10-mV increments (Figure 6D). Venom (100 μg/ml) completely inhibited central pore currents recorded from wild type OtNa_V_1.8 channels but failed to affect background leak currents (Figure 6E). Venom (100 μg/ml) only partially inhibited central pore currents recorded from OtNa_V_1.8 mutant channels, suggesting that the DI S4 double mutation (R215G/R218G) could reduce venom binding affinity towards OtNa_V_1.8 channels (Figure 6F). Venom also significantly depressed inward gating pore currents through the DI voltage sensing domain, suggesting that venom might be a DI S4 gating modifier. To further examine the effects of venom on the DI S4 voltage sensor, we made a single DI mutant by mutating only the first GC R215G. Na^+^ currents were elicited by a 50-millisecond depolarization to +20 mV from a holding potential of −80 mV before and after application of venom (10 μg/ml). After mutating the first GC, venom could no longer inhibit the OtNa_V_1.8 channel (R215G, *n* = 3 cells) (Figure 7). These results suggest that the DI voltage sensor is critical for the inhibitory effects of venom. However, they also raise an interesting question regarding differences in the inhibitory effects of venom on single (first GC) and double (first and second GC) voltage sensor mutants. It is possible that the DI S4 second GC allosterically alters the conformation of the first gating charge, which in turn enhances interaction with venom toxins.

**FIGURE 8:**
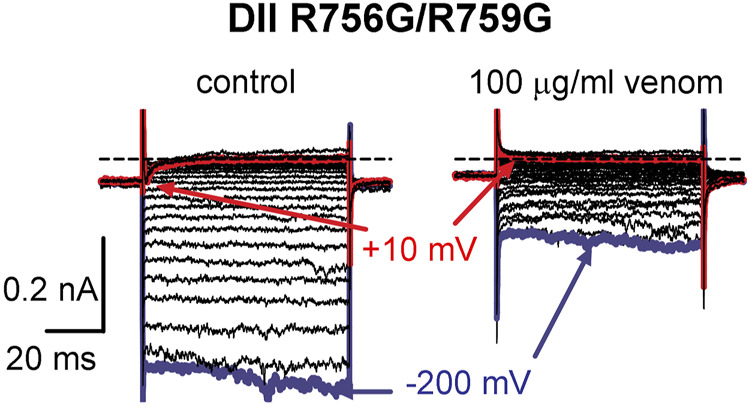
Effects of AZ bark scorpion venom on DII inward gating pore currents. Gating pore currents were generated by glycine mutation of the first and second gating charges (two outermost arginine residues) on OtNa_V_1.8 DII S4 (R756G/R759G). A typical protocol was used to elicit inward gating pore current (see schematic, Figure 6D). Cells held at −80 mV were stimulated by 50-ms hyperpolarizing steps to various potentials that ranged from –200 to +40 mV in 10-mV increments. As observed for wildtype OtNa_V_1.8 channels, venom completely inhibited OtNa_V_1.8 DII S4 R756G/R759G mutant channels, suggesting that the mutations did not affect venom binding affinity. Blue and red traces represent typical leak current (gating pore current) and central pore current at −200 and −10 mV, respectively.

To determine if the DII S4 voltage sensor plays a role in venom inhibition, we mutated the first and second GC R756G/R759G. Venom (100 μg/ml) completely inhibited OtNa_V_1.8 DII R756G/R759G mutant channels, suggesting that the mutation of the two outer most GC in the DII S4 voltage sensor did not affect venom binding affinity (Figure 8). However, while we show that venom altered gating pore currents generated by the DII voltage sensor mutant R756G/R759G, we did not show if the double mutation altered the binding of venom peptides to OtNa_V_1.8. Thus, we cannot completely rule out that the DII voltage sensor may contribute to interaction between venom peptides and OtNa_V_1.8.

**FIGURE 9:**
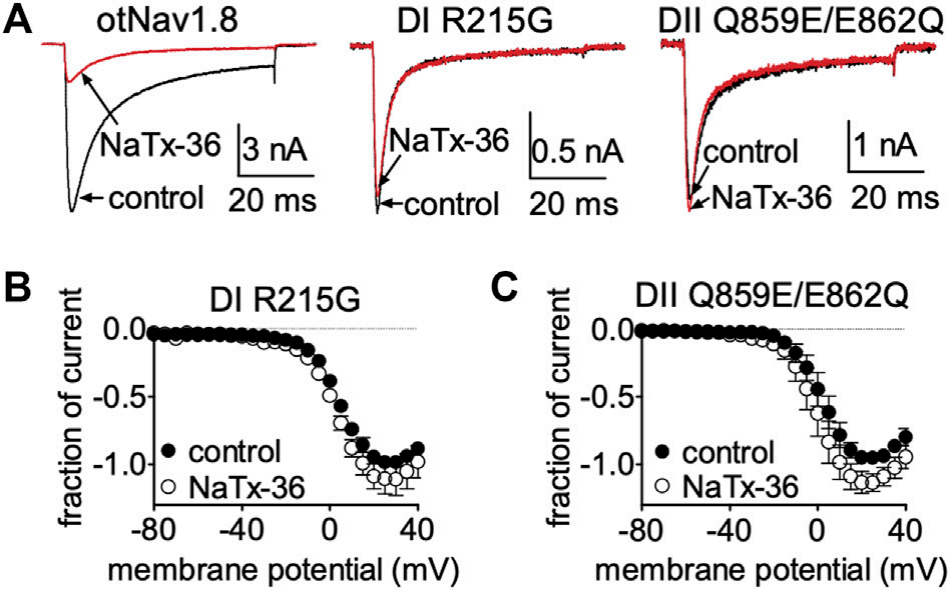
Effects of NaTx36 on DI S4 voltage sensor and DII SS2 – S6 pore loop mutants. **(A)** Representative Na^+^ current traces were elicited by a 50-ms depolarization to +20 mV from a holding potential of −80 mV before and after application of NaTx36 to ND7/23 cells transfected with either DI or DII mutant OtNav1.8. **(B,C)** To determine the fraction of current after toxin treatment, Na^+^ currents were elicited by 50-ms depolarizing steps to various voltages ranging from −80 to +40 mV in 5-mV increments. All Na^+^ currents were normalized to the maximum amplitude of control peak current. **(B)** NaTx36 (100 nM) did not inhibit the OtNa_V_1.8 DI voltage sensor S4 R215G mutant (control, *n* = 3; NaTx36, *n* = 3; *p* = 0.23). **(C)** In addition, NaTx36 (100 nM) had no effect on the DII SS2 – S6 pore loop Q859E/E862Q mutant (control, *n* = 5; NaTx36, *n* = 5; *p* = 0.08). All cells were pretreated with 500 nM TTX to block TTX-sensitive sodium channels.

Collectively, these results suggest that venom peptides may be inhibiting OtNa_V_1.8 via interactions with the first GC (R215) in the DI S4 voltage sensor, and with the acidic residue (E862) in the DII SS2 – S6 pore loop. To determine if these amino acids are also critical for the inhibitory effects of NaTx36, we applied the toxin to the OtNa_V_1.8 DI S4 voltage sensor and DII SS2 – S6 pore loop mutants. Cells were pretreated with 500 nM TTX to block TTX-sensitive sodium channels. Representative Na^+^ current traces were elicited by a 50-ms depolarization to +20 mV from a holding potential of −80 mV before and after application of NaTx36 to ND7/23 cells transfected with either wildtype, DI S4 (R215), or DII SS2 – S6 (Q859E/E862Q) mutant OtNav1.8 (Figure 9A). To determine the fraction of current after toxin treatment, Na^+^ currents were elicited by 50-ms depolarizing steps to various voltages ranging from −80 to +40 mV in 5-mV increments. All Na^+^ currents were normalized to the maximum amplitude of control peak current. NaTx36 (100 nM) failed to inhibit TTX-R Na^+^ currents recorded from the DI voltage sensor mutant (R215G) channels. The fraction of current was 110.9% ±11.1% of control current (control, *n* = 3; NaTx36, *n* = 3; *p* = 0.23) (Figure 9B). In addition, NaTx36 (100 nM) failed to inhibit TTX-R Na^+^ currents recorded from the DII SS2 – S6 pore loop (Q859E/E862Q) mutant channels. The fraction of current after toxin treatment was 136.0% ± 19.7% of control current (control, *n* = 5; NaTx36, *n* = 5; *p* = 0.08) (Figure 9C). These results suggest that the DI S4 first GC R215 and the acidic residue (E862) in the DII SS2 – S6 pore loop are critical for NaTx36 inhibition of OtNa_V_1.8.

**FIGURE 10:**
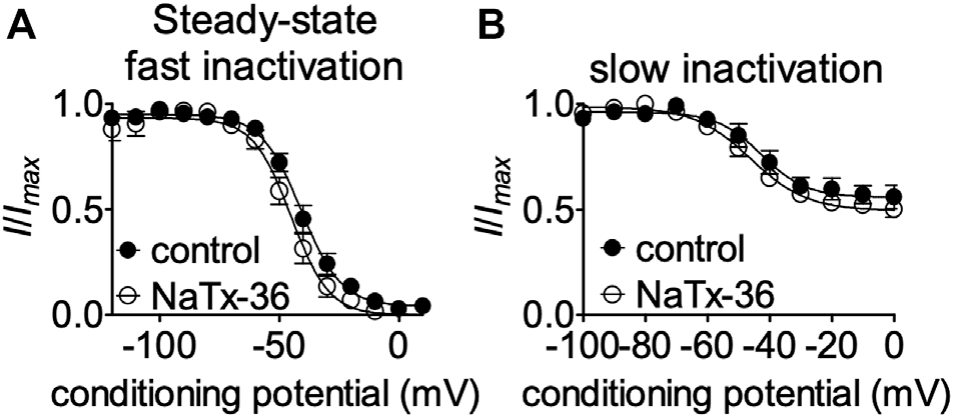
Effects of DI S4 mutation R215G on steady-state fast inactivation and slow inactivation. **(A,B)** Representative steady-state fast inactivation and slow inactivation curves before and after 100 nM NaTx36 application to ND7/23 cells transfected with OtNa_V_1.8 DI S4 first GC R215G mutant channels. Cells were pretreated with 500 nM TTX to block TTX-sensitive sodium channels. All Na^+^ currents were normalized to the maximum amplitude of control peak current. **(A)** Steady-state fast inactivation was measured by a standard double-pulse protocol in which sodium currents were elicited by a 20-ms depolarizing potential of +30 mV following a 500-ms prepulse at voltages ranging from −120 to +10 mV. 100 nM NaTx36 did not affect steady-state fast inactivation of the R215G mutant channels (control, *n* = 8; NaTx36, *n* = 5). **(B)** Steady-state slow inactivation was measured with 5 s pre-pulses ranging from −130 to +10 mV, followed by 10 ms pulses to −80 mV to allow recovery from fast inactivation followed by a 20-ms depolarizing potential of +30 mV. 100 nM NaTx36 did not affect steady-state fast inactivation of the R215G mutant channels (control, *n* = 5 cells; NaTx36, *n* = 4 cells).

NaTx36 shifts the voltage-dependence of steady-state fast inactivation and slow inactivation of wildtype OtNa_V_1.8 channels to hyperpolarized membrane potentials (Figure 5). Given that mutation of the DI S4 R215G abolishes the inhibitory effects of NaTx36 on wildtype OtNa_V_1.8, we asked whether NaTx36 could still shift the voltage dependence of steady-state fast inactivation and slow inactivation of DI S4 R215G mutant channels. To test this, ND7/23 cells were transfected with DI S4 R215G mutant clones. Steady-state fast inactivation was measured by a standard double-pulse protocol in which sodium currents were induced by a 20-ms depolarizing potential of +30 mV following a 500-ms prepulse at voltages ranging from −120 to +10 mV. NaTx36 (100 nM) failed to shift the voltage dependence of steady-state fast inactivation for the R215G mutant channels (control, *n* = 8; NaTx36, *n* = 5) (Figure 10A). Steady-state slow inactivation was measured with 5 s pre-pulses ranging from −130 to +10 mV, followed by 10 ms pulses to −80 mV to allow recovery from fast inactivation followed by a 20-ms depolarizing potential of +30 mV NaTx36 (100 nM) also failed to shift the voltage dependence of steady-state slow inactivation for the R215G mutant channels (control, *n* = 5; NaTx36, *n* = 4) (Figure 10B). These data suggest that the DI S4 R215 is critical for the effects of NaTx36 on OtNa_V_1.8 steady-state fast inactivation and slow inactivation. The data also suggest a link between toxin-induced Na^+^ current inhibition and channel inactivation mechanisms.

### Computational Models: Residues in the DI S1 – S2 and S3 – S4 Linkers, and in the DII S5 – SS1 Pore Loops Are Critical for NaTx36 – OtNa_V_1.8 Interactions

Electrophysiological analyses of OtNa_V_1.8 DI and DII mutant channels suggest that residues in the DI S4 voltage sensor (R215) and DII pore loop SS2 – S6 (E862) are critical for the inhibitory effects of both AZ bark scorpion venom and NaTx36. To further investigate the molecular determinants underlying NaTx36 inhibition of OtNa_V_1.8 Na^+^ current, we computationally modelled the toxin protein bound to grasshopper mouse OtNa_V_1.8.

**FIGURE 11:**
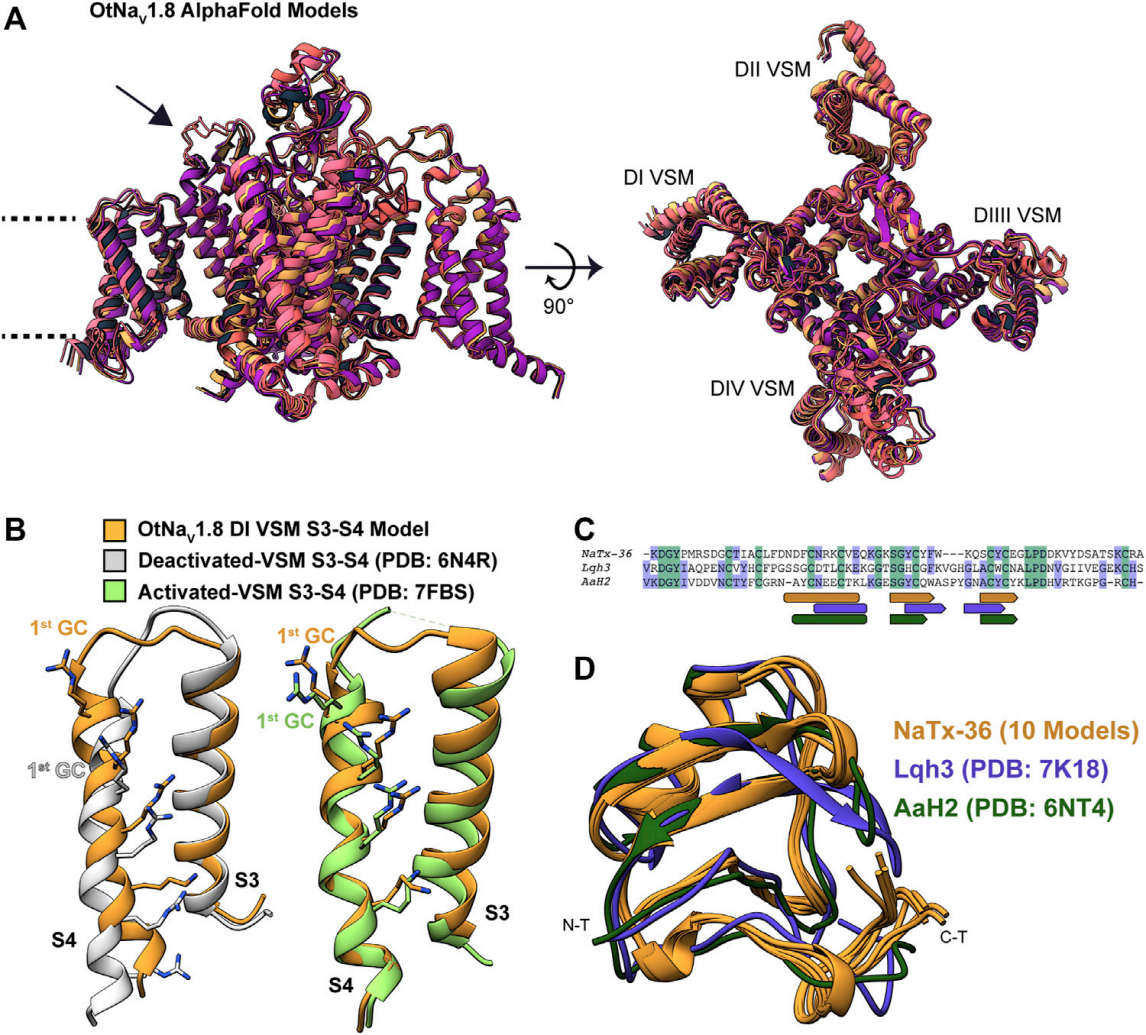
Computational modeling of OtNa_V_1.8 and NaTx36 structures. **(A)** Representative ribbon structures for the five top models of OtNa_V_1.8 generated by AlphaFold; dashed lines represent approximate membrane boundaries, and the black arrow points to the DII S5 – SS1 loop. **(B)** Position of gating charges (GC) within the DI voltage sensor S4 segment of the OtNa_V_1.8 model. DI S3 – S4 segments from the OtNa_V_1.8 model are compared with experimental structures of activated and deactivated voltage sensor segments to illustrate that the OtNa_V_1.8 DI S3 – S4 was modelled in the activated (outward) state. In the activated state, the first GC R215 is exposed to the extracellular solvent. **(C)** Sequence alignment of NaTx36 with two other scorpion toxins, Lqh3 and AaH2, whose biological actions on voltage-gated sodium channels are well-described; rounded rectangles indicate sequence segments corresponding to alpha-helices, and pentagons indicate beta strands. **(D)** NaTx36 models generated by AlphaFold and RosettaFold are superimposed onto the experimental structures of scorpion toxins Lqh3 and AaH2.

Modeling OtNa_V_1.8 channel: We used AlphaFold to model the OtNa_V_1.8 channel based on Na_V_1.8 sequence from grasshopper mice (Onychomys torridus, GenBank: KF717604.1) (Figure 11A). The five predicted structures showed remarkable agreement [root mean square deviation (RMSD) between backbone atoms = 0.6–0.8 Å] apart from conformational differences corresponding to the DII pore module (Figure 11A **, arrow**). The electrophysiological data from the OtNa_V_1.8 DI S4 mutant (R215G) showed that the first GC R215 is critical for NaTx36 inhibition of OtNa_V_1.8 (Figure 9B). This suggests that GC R215 in OtNa_V_1.8 is in the activated (outward) position where it could be exposed to the toxin. We compared the relative position of GC R215 in the OtNa_V_1.8 DI S4 segment with Protein Data Bank (PDB) experimental structures of deactivated (PDB: 6N4R) and activated (PDB: 7FBS) voltage sensors and confirmed that the position of the first GC R215 in the OtNa_V_1.8 DI S4 voltage sensor was modelled in the outward conformation (Figure 11B).

Modeling NaTx36 toxin: We used RosettaFold (five models) and AlphaFold (five models) to generate models of NaTx36. The ten models demonstrated outstanding agreement [root mean square deviation (RMSD) between backbone atoms = 0.2–0.7 Å] with only minor differences in the carboxyl terminal (C-T) region (Figures 11C,D) that are most likely due to conformational flexibility of this region. We compared the predicted fold of NaTx36 with the experimental structures of scorpion toxins Lqh3 (Leiurus quinquestriatus) and AaH2 (Androctonus australis) since their mechanisms of action on Na_V_ channels are structurally well-described (Clairfeuille et al., 2019; Jiang et al., 2021). Sequence identity between NaTx36 and Lqh3 is 29.85%, and between NaTx36 and AaH2 is 34.33% (Figure 11C). The overall structural folds of NaTx36, Lqh3, and AaH2 are remarkably similar; only differing slightly in the position and length of the secondary structure elements (Figure 11D). These differences highlight the ability of scorpions to produce functionally diverse peptide toxins from a structural core that is stabilized by multiple disulfide bonds. This unique structure-function relationship allows a large degree of sequence sampling without disrupting the original folding pattern. Notably, Lqh3, AaH2, and other scorpion peptide toxins have been reported to interact with the DIV S4 voltage sensor in sodium channels (Possani et al., 1999; Possani et al., 2000) while our experimental results suggest that NaTx36 inhibits OtNa_V_1.8 Na^+^ current through manipulation of the DI S4 voltage sensor.

Determination of amino acids in OtNa_V_1.8 critical for binding toxin NaTx36. NaTx36 modifies OtNa_V_1.8 gating (Figures 4, 5), which suggests that NaTx36 inhibits OtNa_V_1.8 by manipulating a channel voltage sensor. Electrophysiological characterization of the OtNa_V_1.8 DI S4 voltage sensor mutant showed that the first GC R215 is critical for the inhibitory effects of both venom and toxin NaTx36 (Figures 6–9). To test the hypothesis that residues in the DI voltage sensor interact with NaTx36, we conducted local ensemble docking using RosettaDock4.0 (Marze et al., 2018) by placing NaTx36 in different initial positions proximal to the OtNa_V_1.8 DI voltage sensing region. This docking protocol is a multi-scale Monte-Carlo based algorithm in which toxin protein properties (translation, rotation, backbone torsional angles, side chain rotamers) are randomly perturbed while multiple conformational ensembles from both the channel and the toxin are queried. Progression through sample space via Monte-Carlo search is scored using a predefined energy function. Perturbations are either accepted or rejected using the Metropolis criterium.

**FIGURE 12:**
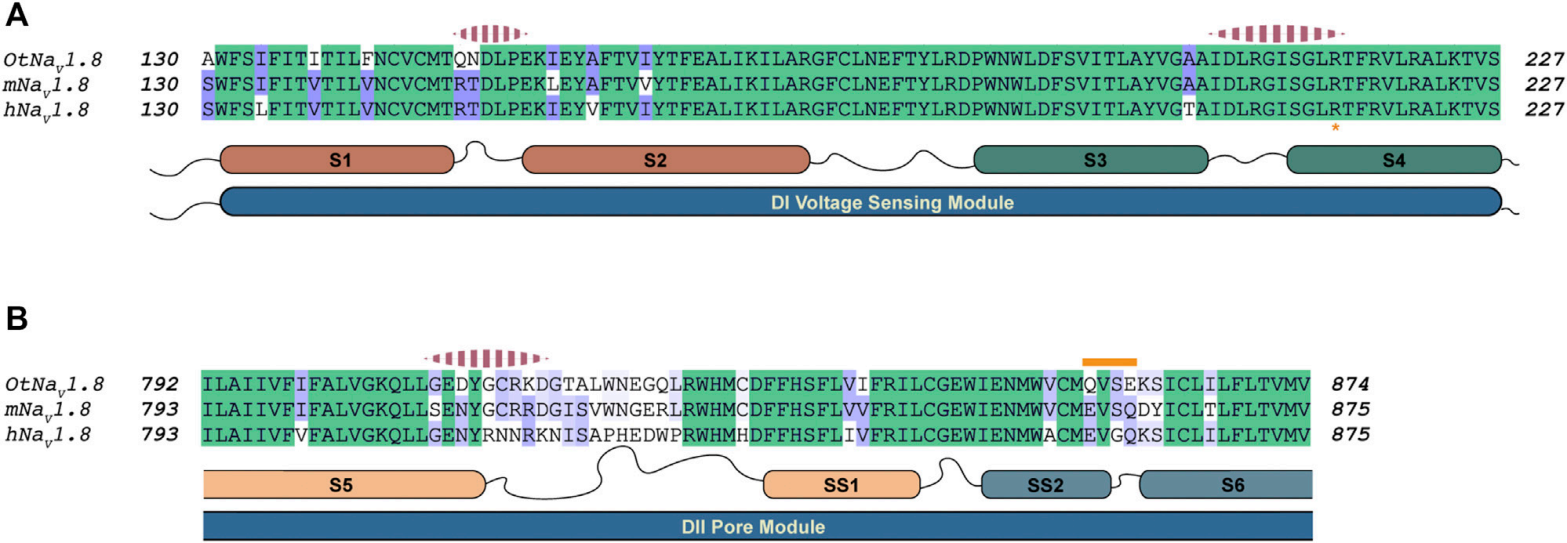
Alignment of Na_V_1.8 from grasshopper mice, house mice, and humans comparing predicted interaction regions between Na_V_1.8 and NaTx36. Sequence from grasshopper mouse (OtNa_V_1.8) DI voltage sensing module (S1 – S2 and S3 – S4) and DII pore module (S5 – SS1, SS2 – S6) was aligned with house mouse (mNav1.8) and human (hNav1.8) sequences to identify motifs uniquely present in OtNa_V_1.8. The alignment color coding is based on sequence identity (high, green; medium, purple; low, white). Regions of the channel that were identified by docking simulations to interact with the toxin are highlighted with pink dashed segments above the corresponding channel sequence. **(A)** Sequence alignment DI voltage sensing module; Orange asterisk at the bottom of the alignment highlights the first gating charge within the S4 segment. **(B)** Sequence alignment corresponding to the second Pore module. The orange bar above the sequence highlights the QVSE motif necessary for NaTx36 inhibitory effect on OtNa_V_1.8.

After generating approximately 20,000 models, we observed that the models converged toward an interface energy minimum (Supplementary Figure S11) suggesting they had reached a near-native binding conformation between NaTx36 and OtNa_V_1.8. By analyzing the binding interface of top scoring models, we identified three regions of the channel interacting with the toxin (Figure 12). Regions one and two correspond to the DI voltage sensing module (S1 – S4) (Figure 12A). Within the voltage sensing module, the OtNa_V_1.8 DI S1 – S2 linker has a unique QN (glutamine, asparagine) motif that is replaced by an RT (arginine, threonine) motif at corresponding positions in house mouse and human Na_V_1.8 (Figure 12A). In grasshopper mice, the QN motif represents two polar uncharged residues with large side chains that replace the positively charged R and polar uncharged T (small side chain) in house mice and human Nav1.8. These changes in grasshopper mice would alter the surface charge and conformation of the DI S1 – S2 linker. The second binding interface was identified in the DI S3 – S4 linker near the extracellular end of the S4 segment (Figure 12A). This region is highly conserved among grasshopper mice, house mice and humans. The third binding interface was identified within the DII pore module (S5 – SS1, SS2 – S6) (Figure 12B). Proximal to the extracellular end of the DI voltage sensing module (Figure 11A **, black arrow**), contacts are identified in the DII S5 – SS1 loop (Figure 12B). This region shows a large degree of sequence variability among grasshopper mouse, house mouse and human Na_V_1.8, and it corresponds to the region of the model that differed among AlphaFold predictions (Figure 11A **, black arrow**). These data suggest that this loop might exhibit increased conformational flexibility compared to other structural regions in the channel. Notably, the DII SS2 – S6 QVSE motif that was experimentally determined to be necessary but not sufficient for the inhibitory effects of either venom (Rowe et al., 2013) or NaTx36 (Figure 9C) on OtNa_V_1.8, is located distally from the DII S5 – SS1 interacting region. The NaTx36 – OtNa_V_1.8 model did not predict any contacts between the toxin and amino acids in the DII SS2 – S6 (Figure 12B **, orange bar**). These data suggest that while NaTx36 does not directly contact residues in the DII SS2 – S6, these residues may cause allosteric interactions with the DII S5 – SS1 loop that contribute to OtNa_V_1.8 inhibition.

**FIGURE 13:**
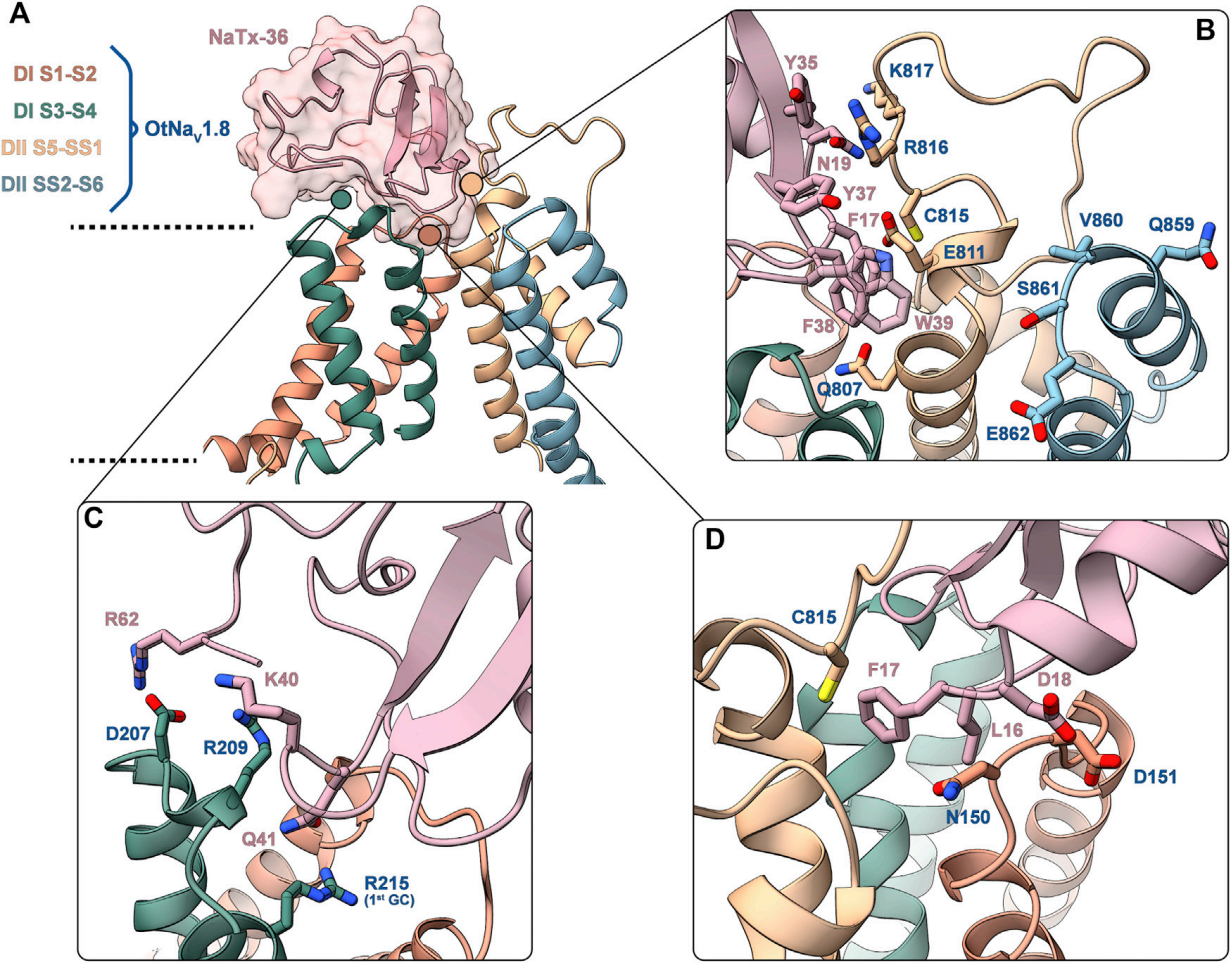
Predicted binding interface between NaTx36 and OtNa_V_1.8. **(A)** Binding interface overview. Dashed lines represent approximate membrane boundaries. Cartoon representation is used for both the channel and the toxin, the molecular surface of NaTx36 is shown with transparency. **(B)** DII S5 – SS1 – NaTx36 interface. QVSE motif within SS2 – S6 is colored blue. **(C)** DI S3 – S4 – NaTx36 interface. **(D)** DI S1 – S2 – NaTx36 interface. Side chains for residues of interest are shown in stick representation.

Further examination of the models of the NaTx36 – OtNa_V_1.8 complex provided important insights into the nature of the interactions between NaTx36 and OtNa_V_1.8 (Figure 13), as well as possible explanations for the importance of the DII SS2 – S6 QVSE motif observed in electrophysiological analyses of OtNa_V_1.8 mutant channels. The models revealed that NaTx36 establishes contacts with channel residues from the three regions described above by adopting a position at the extracellular end of the DI voltage sensing module where the toxin projects its secondary structure elements toward the pore (Figure 13A). This NaTx36 docking pose contrasts with Lqh3 and AaH2 scorpion toxins which bind to Na_V_ DIV S3 – S4 linkers by positioning the beta sheet and alpha-helix away from the pore (Clairfeuille et al., 2019; Jiang et al., 2021).

Interactions with the channel DI S1 – S2 linker (Figure 13D) are established by toxin residues located at the bottom of the toxin alpha-helix. Detailed analysis showed favorable interactions between toxin residues L16, F17 and D18 and channel residue N150 which is uniquely present in OtNa_V_1.8. Notably, Protein Interface Z Score Assessment (PIZSA) software analysis (Roy et al., 2019) of the binding interface revealed that these contacts between the toxin and channel residue N150 account for most of the stabilizing interactions (Supplementary Table S1). Channel residue D151 also interacts with the toxin, although these interactions may contribute less to the binding. Residues L16, F17 and D18 are also utilized by the toxin to engage the DII pore module by strongly interacting with C815.

The OtNa_V_1.8 DI S3 – S4 linker also underlies relevant interactions with charged residues in the toxin (Figure 13C). For example, toxin residue R62, located in the C-terminal tail of NaTx36, interacts with channel residue D207. Toxin residues K40 and Q41 establish a complex set of electrostatic interactions with channel residues R209 in the DI S3 – S4 linker and R215 (first GC) in the DI S4 enabling Q41 to wedge into the voltage sensor to interact with R215. This result might explain why the OtNa_V_1.8 R215G mutant lost sensitivity to NaTx36 inhibition.

In the channel pore module, the DII S5 – SS1 loop interacts with NaTx36 through a set of aromatic (F17, Y35, Y37, F38, W39) and polar uncharged (N19) residues (Figure 13B). Channel residues C815, R816 and K817 interact strongly with toxin residue N19, accounting for the second most important hotspot identified by PIZSA (Supplementary Table S1). In addition, toxin aromatic residues interact with channel residues Q807, E811 and R816, although it is possible that these residues are also important for membrane embedding.

**FIGURE 14:**
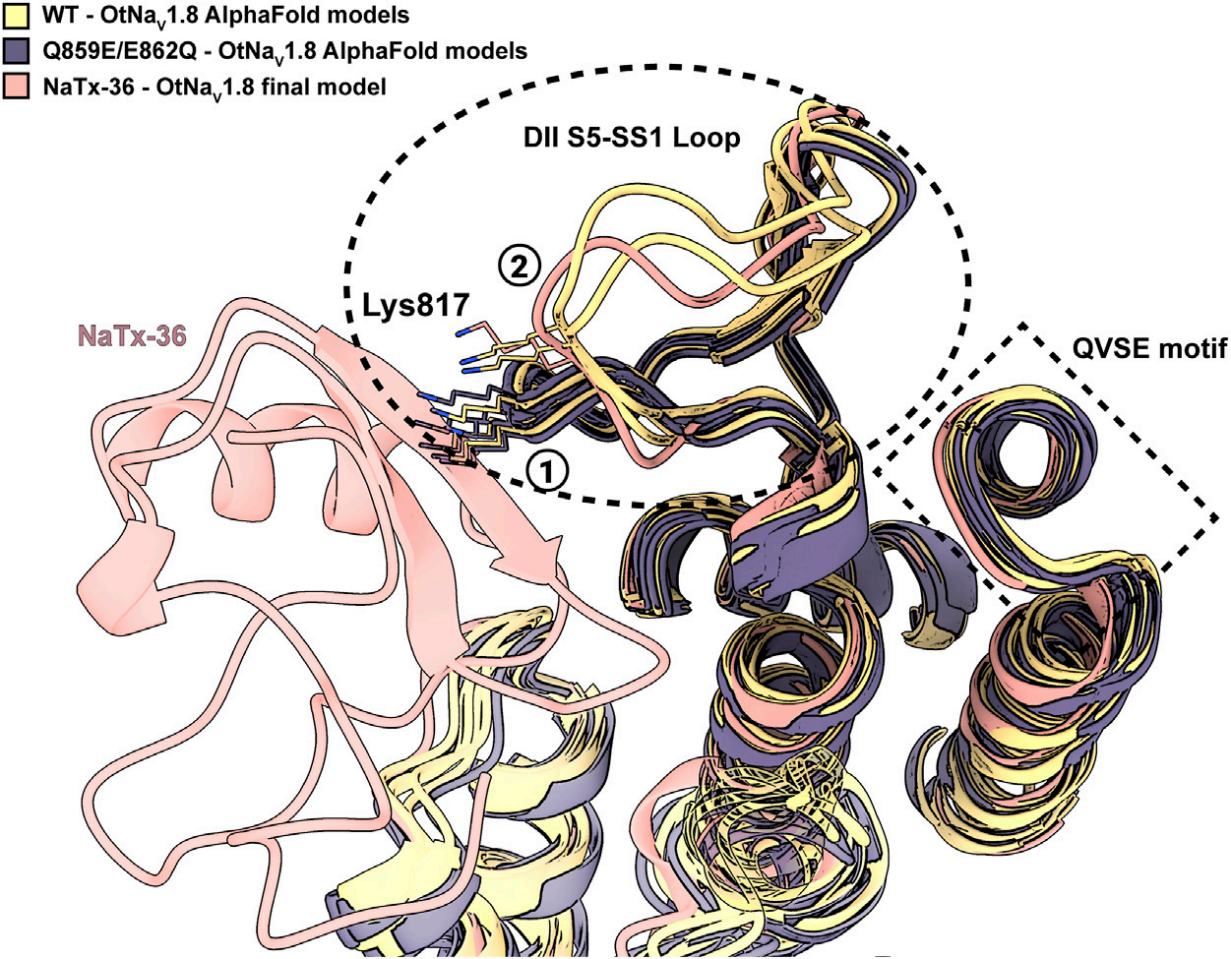
DII S5 – SS1 loop conformations in WT-OtNa_V_1.8 and mutant SS2 – S6 Q859E/E862Q – OtNa_V_1.8. AlphaFold models predict two different conformations for the DII S5 – SS1 loop in the WT channel (see circled 1 and 2); three models in the first conformation and two in the second (yellow models). The second loop conformation is the one predicted by RosettaDock to bind NaTx36 (pink model). However, the second loop conformation is not present in the predicted models for the mutant channel. In the mutant channel SS2 – S6 Q859E/E862Q – OtNa_V_1.8, the DII S5 – SS1 loop was modelled by AlphaFold only in the first conformation observed for the WT (black models). Interestingly, superimposed models predict a steric clash between channel residue Lys817 and NaTx36 if the DII S5 – SS1 loop occupies the first conformation.

To explain the observation that the QVSE motif in the DII SS2 – S6 loop is required for the inhibitory effect of the toxin, we located these residues in our model (Figure 13B, blue segment). We observed that, although these residues are located far from the interface with NaTx36, they closely interact with the DII S5 – SS1 region, which our model predicts is crucial for toxin binding. This observation together with the variability observed in the predicted structure for DII S5 – SS1 loop by AlphaFold, suggests that this loop might have relatively high conformational flexibility. If so, interactions with the QVSE motif in DII SS2 – S6 might modulate the relative occupancy of different conformational states and, thus, affect NaTx36 toxin binding. To test this hypothesis, we used AlphaFold to model OtNa_V_1.8 with the QVSE motif changed to EVSQ (Q859E/E862Q) (Figure 14). We found that AlphaFold predicts two different conformations of the DII S5 – SS1 loop for the WT Na_V_1.8. However, all mutant Q859E/E862Q – OtNaV1.8 models presented the same conformation in this loop. Interestingly, the loop conformation present in our model of NaTx36 bound to WT OtNa_V_1.8 is the one missing in the mutant models. These results suggest a role for the QVSE motif in regulating DII S5 – SS1 loop conformational flexibility. Future experimental testing to confirm functional differences should be conducted to evaluate this hypothesis.

## Discussion

AZ bark scorpion venom inhibits grasshopper mouse Na_V_1.8 Na^+^ current and blocks transmission of pain signals to the brain (Rowe et al., 2013). We fractioned bark scorpion venom and showed that subfraction F11-E inhibits a recombinant grasshopper mouse Na_V_1.8 channel (OtNa_V_1.8) (Mohamed Abd El-Aziz et al., 2021). We chemically synthesized the four peptide toxins (NaTx4, NaTx13, NaTx22, NaTx36) identified from F11-E and tested them on OtNa_V_1.8. Toxin NaTx36 inhibits OtNa_V_1.8 Na^+^ current in a concentration and voltage dependent manner (Figures 2–4). To gain insight into the molecular basis underlying NaTx36 inhibition of Na_V_1.8, we determined the biological action of NaTx36 on OtNa_V_1.8 and identified amino acids that are critical for the toxin-channel interaction complex.

### NaTx36 Enhances Grasshopper Mouse Na_V_1.8 Channel Activation Through a Three-Point Binding Motif

Whole-cell patch clamp electrophysiology demonstrated that NaTx36 lowers the threshold for channel opening by shifting the voltage dependence of activation to hyperpolarized membrane potentials. Current-voltage curves show that in the presence of NaTx36, OtNa_V_1.8 channels begin to open around −60 mV (Figure 4). This suggests that the biological action of NaTx36 is like scorpion β-toxins, which enhance activation through a voltage sensor trapping mechanism. The mechanism proposes that scorpion β-toxins bind Na_V_ at rest and then trap the DII S4 voltage sensor in the activated (outward) position, increasing the probability that channels will open at or near resting membrane potentials (Qu et al., 1998; Cestèle and Catterall, 2000; Catterall et al., 2007). Scorpion β-toxins have a three-point binding site on Na_V_ where the wedge-shaped toxins fit into a solvent accessible cleft formed by the DII voltage sensing module S1 – S2 and S3 – S4 linkers, and the neighboring DIII SS2 – S6 pore loop (Zhang et al., 2011; Zhang et al., 2012). The primary binding site includes residues in the DII S3 – S4 linker that are critical for β-toxins to trap the S4 voltage sensor (Zhang et al., 2011). Secondary binding sites include residues in the DII S1 – S2 linker and the DIII SS2 – S6 pore loop (Zhang et al., 2012). Na_V_ three-dimensional channel structure is organized such that the voltage sensing module (S1 – S4) of each domain is adjacent to the pore module (S5 – S6) of its neighboring domain (domain swapping). Thus, the three points of the DII/DIII β-toxin binding motif are physically close. In contrast to the typical β-toxin DII/DIII binding motif, site-directed mutagenesis and computational models in this study demonstrate that NaTx36 uses residues in the DI voltage sensor and DII pore module to inhibit OtNa_V_1.8. A comparison of gating pore currents from DI S4 (R215G/R218G) and DII S4 (R756G/R759G) double mutant channels suggest that the DI S4 voltage sensor is critical for venom inhibition of OtNa_V_1.8 (Figures 6, 8). A DI S4 (R215G) single mutant channel confirmed that the first GC (R215) in the DI S4 voltage sensor is critical for the inhibitory effects of venom (Figure 7) and NaTx36 (Figure 9). Moreover, the DII mutant channel showed that the pore loop SS2 – S6 Q859E/E862Q motif is important for the inhibitory effects of NaTx36. While the data suggest that NaTx36 employs a DI/DII binding motif as opposed to the characteristic β-toxin DII/DIII motif, the DI/DII reflects a similar domain swapping arrangement that enables the toxin to modulate channel gating via the voltage sensing module.

Computational modeling of the NaTx36 – OtNa_V_1.8 complex supported the experimental electrophysiological data showing that the DI voltage sensor and the DII pore module are critical for NaTx36 activity. However, the models revealed additional details on residues that mediate toxin-channel interactions. First, NaTx36 and OtNa_V_1.8 establish a highly stable complex through interactions between toxin residues L16, F17, and D18 with channel residue N150 in the DI S1 – S2 linker (Figure 13D). NaTx36 also forms electrostatic interactions between toxin residues R62 and K40 and channel residues D207 and R209 in the DI S3 – S4 linker, and toxin residue Q41 with the DI S4 GC R215 (Figure 13C). This motif enables toxin residue Q41 to wedge into the DI voltage sensor to interact with the first GC R215. Further, the models predicted that instead of directly contacting residues in the DII SS2 – S6 pore loop, toxin residues L16, F17, and D18 interact with residue C815 in the channel DII S5 – SS1 pore loop, establishing a link between the toxin and domains DI and DII. Models of the mutant QVSE/EVSQ channel predict that the QVSE motif in the DII SS2 – S6 loop allosterically alters the conformation of the S5 – SS1 loop to enhance toxin-channel interactions. The toxin and channel DI/DII complex may be further stabilized by interactions between a set of aromatic (F17, Y35, Y37, F38, W39) and polar uncharged (N19) residues in NaTx36 and residues C815, R816 and K817 in the channel DII S5 – SS1 pore loop.

Collectively, the site-directed mutagenesis and modeling analyses suggest that NaTx36 enhances activation of OtNa_V_1.8 via a three-point binding motif that includes direct and allosteric interactions with residues in the DI voltage sensing module (S1 – S2 and S3 – S4 linkers, S4 segment) and the DII pore module (S5 – SS1 and SS2 – S6 loops). This motif suggests a potential mechanism for NaTx36 to dock with OtNa_V_1.8 and then trap the DI S4 voltage sensor in the outward position, enhancing channel activation. While the majority of scorpion β- and α-toxin studies suggest that voltage sensors in DII and DIV, respectively, are the primary targets, other systems provide examples of how toxin binding/activity relationships involve additional channel domains. For example, Xiao et al. showed that the spider toxin ProTx-II interacts with a sodium channel DI voltage sensor to modify the voltage dependence of gating pore currents (Xiao et al., 2014). However, the DII S4 voltage sensor was the most critical sensor for interactions with ProTx-II. Using cryo-EM, Clairfeuille et al. discovered that the scorpion α-toxin AaH2, which blocks fast inactivation, binds a site in the DI voltage sensing module in addition to its high affinity binding site in the DIV voltage sensor (Clairfeuille et al., 2019). Given that NaTx36 hyperpolarizes the voltage dependence of OtNa_V_1.8 steady-state fast inactivation, and DIII and DIV play a role in fast inactivation, future work on the NaTx36 – OtNa_V_1.8 complex should investigate the effects of NaTx36 on the DIII and DIV voltage sensors.

### NaTx36 Modifies Grasshopper Mouse Na_V_1.8 Fast Inactivation Gating

Na_V_ S4 voltage sensors respond to depolarizing membrane potentials by moving outward. Movement of the DI – DIII voltage sensors combined with partial movement of the DIV voltage sensor initiates opening of the channel activation gate; movement of the DIV voltage sensor triggers fast inactivation gating (Armstrong, 2006; Gilchrist and Bosmans, 2018). As described above, NaTx36 enhances OtNa_V_1.8 opening by lowering the threshold for channel activation. In addition, NaTx36 modifies steady-state fast inactivation gating in OtNa_V_1.8 by substantially shifting the voltage dependence to hyperpolarized potentials (Figure 5A). Notably, few scorpion toxins have been characterized in terms of their effects on the voltage dependence of fast inactivation gating. Typically, scorpion toxins are characterized regarding effects on the voltage dependence of activation (β-toxins trap the DII voltage sensor in the outward position, shifting activation to hyperpolarized potentials that open channels) or effects on fast inactivation kinetics (α-toxins trap the DIV voltage sensor in the inward position, delaying fast inactivation gating) (Qu et al., 1998; Cestèle and Catterall, 2000; Catterall et al., 2007; Campos et al., 2008; Clairfeuille et al., 2019). However, the relationship between activation and inactivation gating, and how toxins alter the voltage dependent properties of that relationship, can provide insight into channel availability. For example, the scorpion β-toxin BMK I shifts the voltage dependence of both activation and fast inactivation gating to hyperpolarized potentials in rat Na_V_1.8, causing an increase in small diameter DRG TTX-R Na^+^ current and pain-related behavior (Ye et al., 2015). Similarly, the recently described tarantula toxin β-theraphotoxin-Eo1a activates Na_V_1.8 and causes pain in house mice by hyperpolarizing activation and steady-state fast inactivation (Deuis et al., 2021). The DIV voltage-sensor-binding toxins Hm1a and LqqIV shift the voltage dependence of Na_V_1.1 fast inactivation gating to depolarized potentials, increasing channel availability and window currents (Osteen et al., 2017). These window currents were attributed to toxin effects on fast inactivation gating as opposed to delayed inactivation kinetics. In contrast, the cobra toxin Na1a inhibits rat Na_V_1.8 activity by depolarizing activation and hyperpolarizing fast inactivation (Zhang et al., 2019). Collectively, these studies highlight the importance of examining the effects of toxins on the voltage dependence of activation and inactivation gating to determine how toxins alter channel excitability. Further examination of the effects of NaTx36 on OtNa_V_1.8 activation and fast inactivation gating will be critical for estimating channel availability, and for determining whether decreased availability underlies NaTx36 inhibition of OtNa_V_1.8.

### NaTx36 Modifies Grasshopper Mouse Na_V_1.8 Slow Inactivation Gating

In addition to modifying activation and fast inactivation gating, NaTx36 modifies slow inactivation gating in OtNa_V_1.8 by substantially shifting the voltage dependence to hyperpolarized membrane potentials (Figure 5B). Slow inactivation differs from fast inactivation in voltage- and time-dependent properties. While fast inactivation inhibits Na_V_ excitability within milliseconds of action potential firing, slow inactivation develops in response to prolonged membrane depolarization (tens of seconds) or a series of action potentials, inhibiting Na_V_ excitability over longer timescales (seconds to minutes) (Vilin and Ruben, 2001; Ulbricht, 2005; Gawali et al., 2016; Ghovanloo et al., 2016). Transition into the slow inactivated state is characterized by a decrease in Na^+^ current amplitude due to a reduction in the population of channels available to fire action potentials. The number of channels rendered unavailable during slow inactivation is proportional to the duration and frequency of the membrane depolarizations that initiate slow inactivation. For example, Blair and Bean (Blair and Bean, 2003) demonstrated that nociceptive sensory neurons transitioned into the slow inactivated state following prolonged electrical and chemical stimulation. Sustained excitation of DRG membranes by either electrical stimulation or capsaicin caused a decrease in Na_V_1.8 TTX-R Na^+^ currents and action potential firing. More recently, Zhang and Bean showed that cannabidiol (CBD) inhibited house mouse Na_V_1.8 in a state-dependent manner that decreased channel availability (Zhang and Bean, 2021). During depolarizing potentials, CBD induced fast binding to inactivated channels, while repolarizing potentials induced slow unbinding from channels. Zhang and Bean concluded that CBD reduces repetitive action potential firing and DRG neuronal excitability by enhancing the slow inactivated state of Na_V_1.8. Thus, the hyperpolarizing effects of NaTx36 on OtNa_V_1.8 slow inactivation may contribute to inhibition of Na^+^ current by reducing the population of available channels. Given that NaTx36 also hyperpolarizes OtNa_V_1.8 activation and fast inactivation, it is plausible that the relationship between activation and fast inactivation gating prolong the excitability of OtNa_V_1.8 or induce repetitive action potentials, triggering channel transition to a slow inactivated state. Moreover, to reverse slow inactivation, membranes must be hyperpolarized. Holding OtNa_V_1.8 at hyperpolarized potentials reduced the inhibitory effects of NaTx36 (Figure 4). Further examination of activation-inactivation (fast and slow) gating relationships, and how toxins alter those relationships, will be critical for characterizing the mechanisms underlying NaTx36 inhibition of OtNa_V_1.8.

### Summary and Conclusion

In primary sensory nociceptive neurons, Na_V_1.8 is crucial for transmitting pain signals to the brain. Given that Na_V_1.8 is linked to neuropathic and inflammatory pain, it has the potential to serve as a drug target. However, the mechanisms that regulate Na_V_1.8 gating are not completely understood. While animal-derived toxins have provided tools for examining structure-activity relationships in several Na_V_, fewer toxins have been identified that modify Na_V_1.8 gating (Ye et al., 2015; Zhang et al., 2019; Deuis et al., 2021). AZ bark scorpions produce venoms rich in peptide toxins that modify the gating mechanisms of Na^+^ ion channels in nerve and muscle tissue (Jaimovich et al., 1982; Couraud et al., 1984; Possani et al., 1999; Corona et al., 2001; Rodríguez de la Vega and Possani, 2005; Carcamo-Noriega et al., 2018). Toxins induce extreme pain in sensitive animals (Rowe et al., 2011; Rowe et al., 2013; Niermann et al., 2020). Southern grasshopper mice prey on bark scorpions, having evolved reduced sensitivity to pain-inducing toxins via amino acid substitutions in Nav1.8 (Rowe and Rowe, 2006; Rowe and Rowe, 2008; Rowe et al., 2013). In this study, we tested four synthetic versions of peptide toxins (NaTx4, NaTx13, NaTx22, NaTx36) identified from a subfraction of AZ bark scorpion venom that inhibited a recombinant grasshopper mouse Na_V_1.8 channel (OtNa_V_1.8). Of the four peptides, NaTx36 inhibited OtNa_V_1.8 in a concentration and voltage dependent manner recapitulating the effects of venom. Interestingly, NaTx36 hyperpolarized the voltage dependence of OtNa_V_1.8 activation, fast inactivation, and slow inactivation gating. Site-directed mutagenesis and computational modeling demonstrated that NaTx36 interacts with OtNa_V_1.8 via amino acids in the DI voltage sensor and DII pore module, as opposed to most scorpion β-toxins that interact with Na_V_ via residues in the DII voltage sensor and DIII pore module. Thus, interactions between NaTx36 and OtNa_V_1.8 provide a novel system for investigating links between activation – inactivation gating relationships, Na_V_ channel availability, and mechanisms that inhibit Na_V_1.8 activity and pain-related behavior. Moreover, NaTx36 may serve as a template for structure-guided development of Na_V_ targeting peptides to treat pain without addiction (Nguyen and Yarov-Yarovoy, 2022).

## Methods and Materials

### Venom Extraction

AZ bark scorpions were collected from the Santa Rita Experimental Range (University of Arizona, Santa Rita Mountains, AZ, United States). Crude venom was extracted from the venom glands using electrical stimulation according to previously published protocols (Clairfeuille et al., 2019). The crude venom samples were hydrated in sterile water, centrifuged, and filtered (0.45 µm sterile filter) to remove insoluble components. Aliquots of the supernatant (hereafter referred to as venom) were lyophilized and stored at −80°C.

### Toxin Peptide Synthesis

Peptide toxins NaTx4, NaTx13, NaTx22, and NaTx36 were chemically synthesized by SB-PEPTIDE (SmartBioscience SAS, France) using the amino acid sequence of peptide primary structures previously determined (Mohamed Abd El-Aziz et al., 2021). Peptides were assembled stepwise using Fmoc-based Solid Phase Peptide Synthesis (SPPS) on a PTI Symphony synthesizer on resin. The Fmoc protecting group was removed using 20% piperidine in DMF and free amine was coupled using tenfold excess of Fmoc amino acids and HCTU/DIEA activation in NMP/DMF (3 × 15 min). Linear peptides were de-protected and cleaved from the resin with a cleavage cocktail, then precipitated out in cold diethyl ether. The resulting white solids were washed twice with diethyl ether and re-suspended. Oxidative folding of the crude linear peptides was conducted at room temperature (RT) in oxidative conditions. Final peptides were purified using Reverse-Phase High Performance Liquid Chromatography (RP HPLC). Samples were injected into a C18 column (150 × 4.6 mm, 130 Å, 2.5 µm) using the following gradient:

**Table:** 

Time (min)	%B
0	5
2	5
22	60
22.1	95
27	95

Buffer A = 0.1% TFA in H20 and Buffer B = 0.1% TFA in acetonitrile. Peptides were controlled by ESI-HRMS and HPLC on Agilent systems, then freeze-dried. SB-PEPTIDE confirmed the mass using Liquid Chromatography Mass Spectrometry (LC MS) (copies of the SB-PEPTIDE Certificate of Analysis, HPLC, and MS data available upon request). Peptides were quantified using OD280 and checked using Nanodrop at 205 nM wavelength. The purity, intact mass and amino acid sequence of each peptide was then validated by co-authors in this study using HPLC, LC MS, and bottom-up MS/MS, respectively (see Sections 4.3 and 4.4 below for method details, see also Supplementary Figures S1–S8).

### LC-MS Validation of Synthetic Toxin Peptides

BioPharma Finder 4.1 (Thermo Scientific) was used for deconvoluting the intact mass spectrum of synthetic peptide toxins (NaTx36, NaTx4, NaTx13, NaTx22), using the Xtract algorithm to calculate the monoisotopic mass. A fit factor of 0.65 was applied together with a signal-to-noise ratio cutoff of 3. Bottom-up proteomics data were searched using the SEQUEST algorithm included in Proteome Discoverer 4.0 (Thermo Scientific) against a custom database containing the non-processed sequence (i.e., signal peptide included) of (NaTx36, NaTx4, NaTx13, NaTx22). The following search parameters were used in SEQUEST: precursor mass tolerance 10 ppm; fragment mass tolerance 0.1 Da; carbamidomethylation as dynamic modification of Cysteines.

### Mass Spectrometry-Base Validation of Synthetic Toxin Peptides

The intact forms of the synthetic peptide toxins NaTx36, NaTx4, NaTx13, and NaTx22 were analyzed after proteolysis to confirm the primary structure and the absence of modifications using liquid chromatography on-line coupled to mass spectrometry (LC-MS). All mass spectrometry measurements were performed on an Orbitrap Eclipse mass spectrometer (Thermo Scientific, San Jose, CA, United States), while liquid chromatography analysis of the whole toxin or its proteolytic peptides was carried out using an Ultimate 3000 UHPLC chromatographic system (Thermo Scientific). For all LC-MS runs, the mobile phases were composed as follows: mobile phase A, 5% acetonitrile (v/v) and 0.2% formic acid (v/v) in water; mobile phase B, 5% water (v/v) and 0.2% formic acid (v/v) in acetonitrile. All solvents were LC-MS grade. For intact mass determination, the synthetic sample was desalted using C4 ZipTips (MilliporeSigma, St. Louis, Missouri) and about 200 ng were loaded onto a nanobore column (100 µm × 200 mm) in-house packed with PLRP-S (Agilent, Santa Clara, California). Separation was conducted using a 45 min gradient, and electrosprayed toxin cations were detected over a 400–2,000 *m*/*z* window using 120,000 resolving power (at *m*/*z* 200), with the Orbitrap Eclipse set in “protein mode”. For bottom-up proteomics, 5 µg of sample were dissolved in 8 M guanidinium chloride and denatured by heating at 100°C for 30 min. Disulfide bond reduction was then performed using 200 mM tris(2-carboxyethyl)phosphine for 30 min at 100°C, and was followed by Cys alkylation incubating the sample with 25 mM iodoacetamide for 20 min at 37°C in the dark. Guanidinium chloride was then diluted to 0.5 M using 100 mM Tris-HCl at pH 8.5 and the sample was digested using 0.4 µg of trypsin for 18 h at 37°C under shaking. Proteolytic peptides were desalted using a C18 spin column (Thermo Scientific) according to the manufacturer’s instructions. LC-MS experiments were based on a commercial trap and analytical nanobore C18 column (Acclaim PepMap, 75 µm × 150 mm, Thermo Scientific) and separation was conducted over a 45 min gradient. The Orbitrap Eclipse was operated in “peptide mode” using a Top-S data-dependent acquisition method (3 s cycle); broadband spectra were collected over a 375–2,000 *m*/*z* window using 120,000 resolving power (at *m*/*z* 200), while peptides were fragmented via higher-energy collisional dissociation (NCE = 35%) and tandem mass spectra were recorded in the Orbitrap at 15,000 resolving power (at *m*/*z* 200). Dynamic exclusion was set to 30 s.

### Culture and Transfection of ND7/23 Cells

Venom and NaTx36 samples were screened for inhibitory activity against a recombinant Nav1.8 clone from grasshopper mice. The gene encoding O. torridus Nav1.8 was inserted into a plasmid with a fluorescent marker (pcDNA3.1-EGFP) for expression in a hybrid cell line (ND7/23). The recombinant Nav1.8 clone is referred to as OtNav1.8. ND7/23 cells were purchased from Novagene [European Collection of Cell Cultures (ECCC), Salisbury, United Kingdom] and cultured under standard conditions according to guidelines provided by the ECCC (37°C in a humidified incubator supplying 5% CO_2_, in Dulbecco’s modified Eagle medium supplemented with 10% fetal bovine serum and 1% penicillin-streptomycin). For patch clamp recording (see below), ND7/23 cells were plated on cover glass chips treated with 0.01% Poly-l-lysine (Sigma, St. Louis, MO, United States). Plated cells were transfected with plasmids encoding α-, β1-, and β2-OtNav1.8 subunits genetically linked to NH2-terminal eGFP using Lipofectamine 3000 reagent (L3000015, Invitrogen, Carlsbad, CA, United States), as described in the manufacturer’s protocol. In brief, 60% confluent cells in a 35-mm dish were treated with 14 μg of total plasmid cDNA for 24–48 h. Cells exhibiting green fluorescence were used for patch clamp recording.

### Electrophysiology Recording

Whole-cell patch clamp electrophysiology was used to record the effects of venom and peptide toxins on OtNa_V_1.8 Na^+^ current expressed in ND7/23 cells. Lyophilized venom and toxin peptide samples were hydrated in sterile, double distilled water to make stock solutions. Concentrations of stock solutions were confirmed using the average of three nanodrop readings. Samples of venom and peptide toxins were then diluted in external bath solution (containing in mM: 140 NaCl, 3 KCl, 1 MgCl_2_, 1 CaCl_2_, and 10 HEPES; pH was adjusted to 7.3 with NaOH) to the desired final concentration. Tetrodotoxin (TTX; 500 nM) was added to the bath solution when recording recombinant OtNa_V_1.8 currents from ND7/23 cells to remove TTX-sensitive Na^+^ currents. The whole-cell membrane currents were recorded at room temperature (21–24°C) using a low noise patch clamp amplifier (Axopatch 200B) interfaced via a Digidata 1550B system to a PC running the pClamp 11 software (Axon Instruments, Molecular Devices, San Jose, CA, United States). All currents were filtered at 1 kHz. Patch pipettes were pulled from borosilicate glass capillaries (World Precision Instruments, Inc., FL, United States) using either a P-97 or P-1000 Flaming/Brown micropipette puller (Sutter Instrument, Novato, CA, United States) and fire-polished on a micro-forge (MF-830; Narishige Scientific Instrument, Japan). The initial resistance was 0.8–1.5 MΩ when filled with the pipette internal solution (containing in mM: 140 CsF, 10 NaCl, 1.1 EGTA, and 10 HEPES; pH adjusted to 7.3 with CsOH). Current traces were evoked by a 100-millisecond depolarizing potential of +20 mV from the holding potential at −80 mV. Current–voltage curves were generated by voltage-clamp protocols consisting of a holding potential of −80 mV followed by a series of 50-ms depolarizations from −80 to +60 mV in 5-mV increments. In the hyperpolarization experiments, the current–voltage curve was generated by voltage-clamp protocols consisting of a holding potential of −120 mV for 30 s followed by a series of 50-ms depolarizations from −80 to +60 mV in 5-mV increments. All venom and venom protein effects were compared to baseline values obtained in vehicle (bath solution) in the same cell. After control responses were obtained, samples of either venom or peptide toxin NaTx36 (concentrations ranging from 0.1 to 25 μg/ml) were added to the chamber (either 87 or 250 µl total volume) and protocols were repeated. The pClamp 11.1 software (Axon Instruments, Molecular Devices) was used for signal acquisition and analysis. The data were filtered at 1 kHz and digitized at 1 kHz using a data acquisition interface 1550B (Axon Instruments, Molecular Devices, San Jose, CA, United States). The whole cell capacitance transient and series resistances were compensated (70%–85%).

### Statistics

Data were analyzed and plotted using ClampFit 11.1 (Molecular Devices), GraphPad Prism 9 (GraphPad Software, Inc., San Diego, CA, United States) and OriginPro 2021b (OriginLab Corp. Northampton, United States). Summarized whole-cell current data are reported as the mean ± SEM of the OtNa_V_1.8 current density. Summarized data were compared using Student’s unpaired *t*-test, with *p* < 0.05 considered significant. The half inhibitory concentration (IC_50_) of NaTx36 on OtNa_V_1.8 channels was estimated by fitting the data to a Dose-response equation with variable Hill slope [y = A1 + (A2 - A1)/(1 + 10^ ((Log X0 – X) * p)) ] where A1 = bottom asymptote, A2 = top asymptote, Log X0 = center, and p = Hill slope.

### Site-Directed Mutagenesis

Site-directed mutagenesis was conducted to introduce mutations into the DI voltage sensor and the DII pore module of the OtNav1.8 recombinant channel. Mutations were introduced using either Agilent QuikChange II (Agilent Technologies, Inc., Santa Clara, CA, United States) or New England Biolabs Q5 (Ipswich, MA, United States) site-directed mutagenesis kits, according to the manufacturer’s protocol. PCR products were used to transform DH5α competent cells (New England Biolabs). Mutations were confirmed using sanger sequencing.

### Primers

R215G forward: 5′-CGA​GGA​ATC​TCA​GGC​CTA​GGG​ACA​TTC​CG-3′
R215G reverse: 5′-CGG​AAT​GTC​CCT​AGG​CCT​GAG​ATT​CCT​CG-3′
R215G/R218G forward: 5′-CCT​AGG​GAC​ATT​CGG​AGT​TCT​CAG​GGC​C-3′
R215G/R218G reverse: 5′-GGC​CCT​GAG​AAC​TCC​GAA​TGT​CCC​TAG​G-3′
E862Q forward: 5′-GGT​CTG​CAT​GCA​AGT​CAG​TCA​GAA​ATC​CAT​CTG​C-3′
E862Q reverse: 5′-GCA​GAT​GGA​TTT​CTG​ACT​GAC​TTG​CAT​GCA​GAC​C-3′
Q859E/E862Q forward: 5′-GGT​CTG​CAT​GGA​AGT​CAG​TCA​GAA​ATC​CAT​CTG​C-3′
Q859E/E862Q reverse: 5′-GCA​GAT​GGA​TTT​CTG​ACT​GAC​TTC​CAT​GCA​GAC​C-3′
R756G/R759G forward: 5′-TCT​GTG​CTT​GGG​ACC​TTC​GGT​TTG​CTG​CG-3′
R756G/R759G reverse: 5′-CGC​AGC​AAA​CCG​AAG​GTC​CCA​AGC​ACA​GA-3′

### OtNa_V_1.8 Modeling

The complete sequence for Na_V_1.8 from grasshopper mice (O. torridus, GenBank: KF717604.1) was trimmed to remove N and C terminal intracellular regions as well as two, large intracellular interdomain loops that are usually not resolved in available experimental structures of mammalian sodium channels. Trimmed sequence was used as input for AlphaFold (Jumper et al., 2021) in Google Colab (Mirdita et al., 2021) with 24 max recycles. Five models of OtNaV1.8 were generated and optimized using Rosetta FastRelax protocol (Conway et al., 2014) and ref2015 energy function (Alford et al., 2017).

### NaTx36 Modeling

NaTx36 amino acid sequence (Mohamed Abd El-Aziz et al., 2021) was used as input for AlphaFold and RosettaFold (Baek et al., 2021) to generate 10 models, 5 with each method. Resulting models were fast relaxed as performed for OtNa_V_1.8 models.

### Computational Docking of NaTx36 to OtNa_V_1.8 DI Voltage Sensing Module

Relaxed models of both toxin and channel were used to generate conformational ensembles as previously described (Marze et al., 2018). NaTx36 was located in three different initial positions and orientations around the OtNa_V_1.8 DI voltage sensing module (S1 – S4) to generate the inputs for RosettaDock4.0 (Marze et al., 2018). Approximately 20,000 docked models were generated and analyzed. Automatic analysis and counting of interacting residues from top scoring models were accomplished by making use of the Protein Interface Z Score Assessment (PIZSA) software (Roy et al., 2019). Selection of the final model was based on the lowest interface score (I_sc).

### Molecular Graphics Visualization

All models were processed and analyzed using UCSF ChimeraX (Goddard et al., 2018).

## Data Availability

The gene encoding Onychomys torridus scn10a and the dataset for the AZ Bark scorpion venom gland transcriptome can be found in the following repositories: https://www.ncbi.nlm.nih.gov/genbank/, KF717604; https://www.ncbi.nlm.nih.gov/genbank/, PRJNA340270. The electrophysiology, mass spectrometry, and computational modeling datasets for this study are available upon request.
