# Supplementary material for: Structural and Functional Characterization of a Novel Scorpion Toxin that Inhibits NaV1.8 via Interactions With the DI Voltage Sensor and DII Pore Module
Source: Front Pharmacol. 2022 May 19;13:846992. doi: 10.3389/fphar.2022.846992 (PMC9160825; doi:10.3389/fphar.2022.846992)
Supplement: Supplementary file 1 [file DataSheet1.PDF]

Supplemental Figures

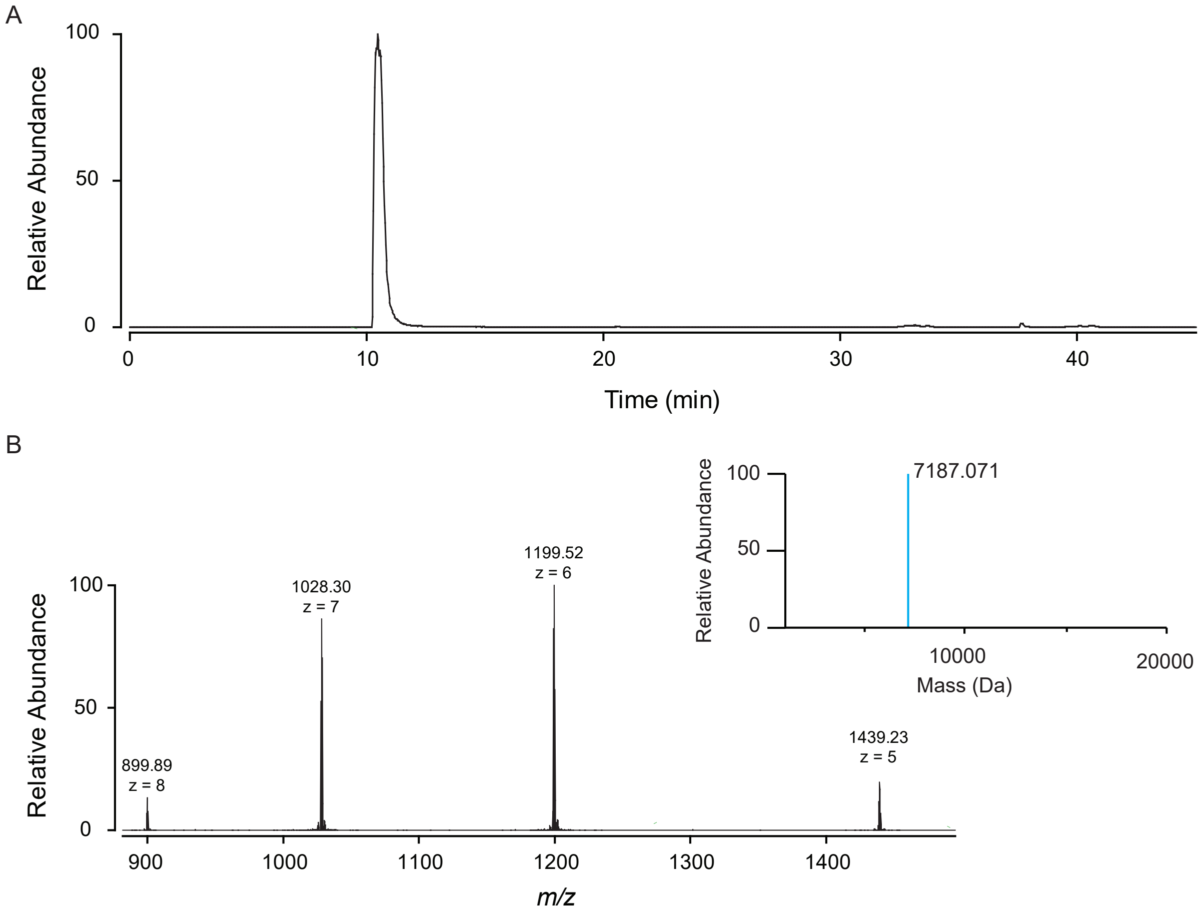

Figure S1. Intact mass analyses of synthetic NaTx36. **(A)** Chromatogram of NaTx36 during intact mass analysis. **(B)** High resolution (120,000 at 200 *m/z*) mass spectrum of the synthetic peptide toxin. Insert shows the deconvoluted mass of NaTx36; calculated mass from spectrum: 7187.071 Da; theoretical mass from sequence: 7187.077 Da; mass error: 0.006 Da (0.8 ppm).

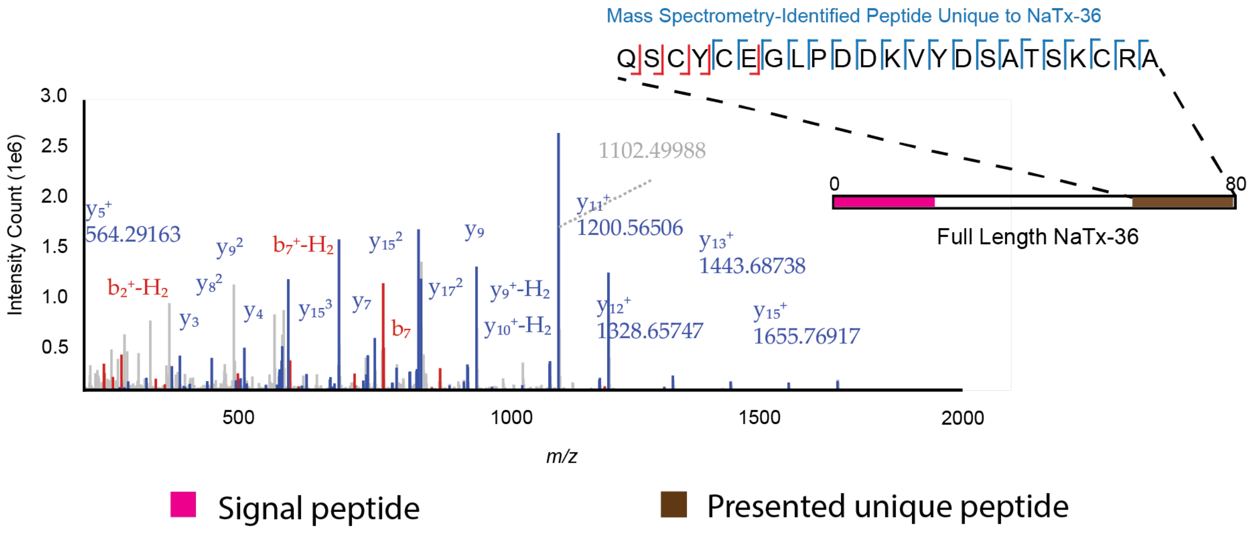

Figure S2. Summary of bottom-up proteomics data from synthetic venom peptide NaTx36. The tandem mass spectrum obtained via higher-energy collisional dissociation is represented with *b*-type product ions indicated in red and *y*-type ions in blue. The inset shows the sequencing of the C-terminal proteolytic peptide, which corresponds to the brown bar in the toxin sequence. The database search used the whole sequence of the toxin, but the signal peptide (indicated in magenta) was not identified, as expected. Total sequence coverage for the mature toxin = 41%, excluding the signal peptide.

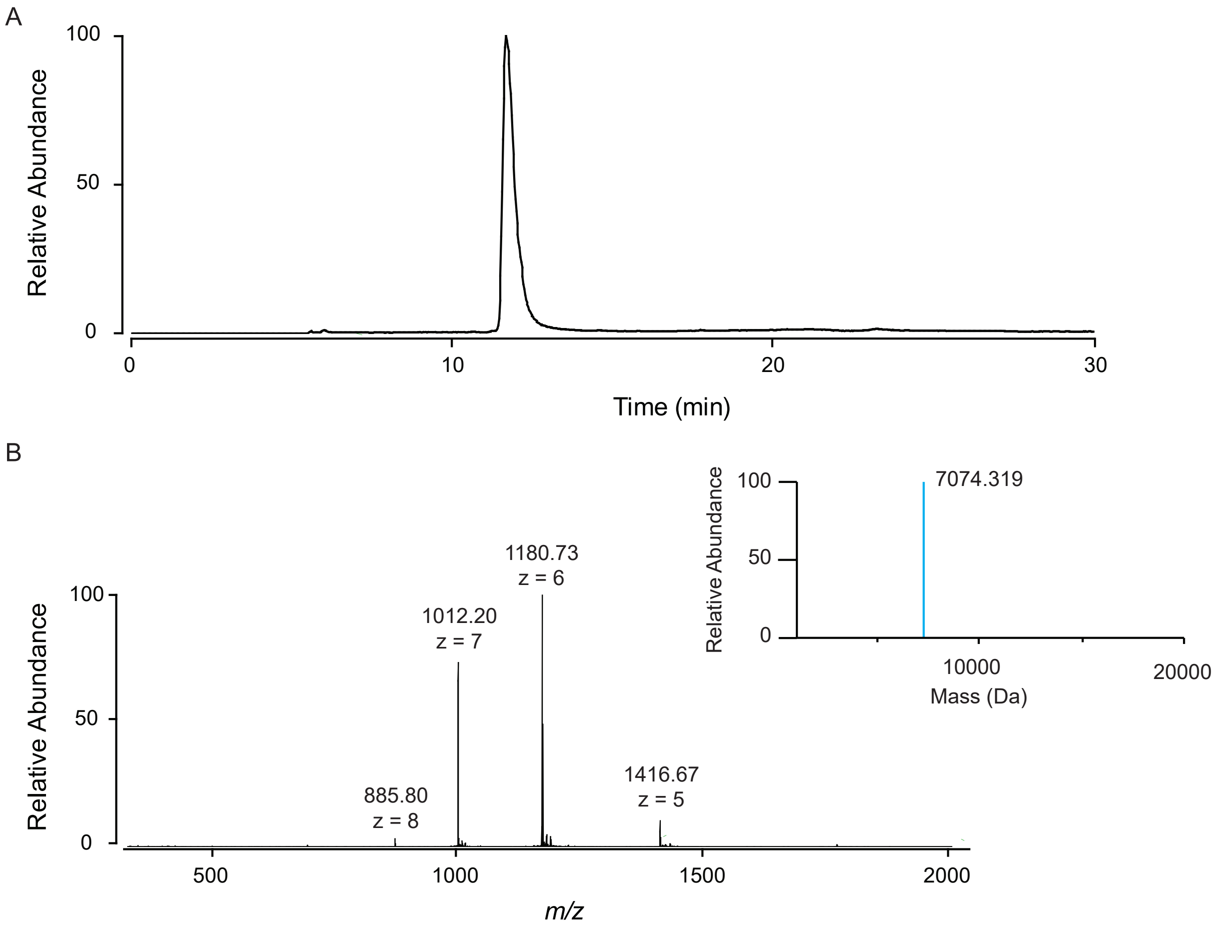

Figure S3. Intact mass analyses of synthetic NaTx4. **(A)** Chromatogram of NaTx4 during intact mass analysis. **(B)** High resolution (120,000 at 200 *m/z*) mass spectrum of the synthetic peptide toxin. Insert shows the deconvoluted mass of NaTx4; calculated mass from spectrum: 7074.319 Da; theoretical mass from sequence 7074.31: Da; mass error: 0.009 Da (0.7 ppm).

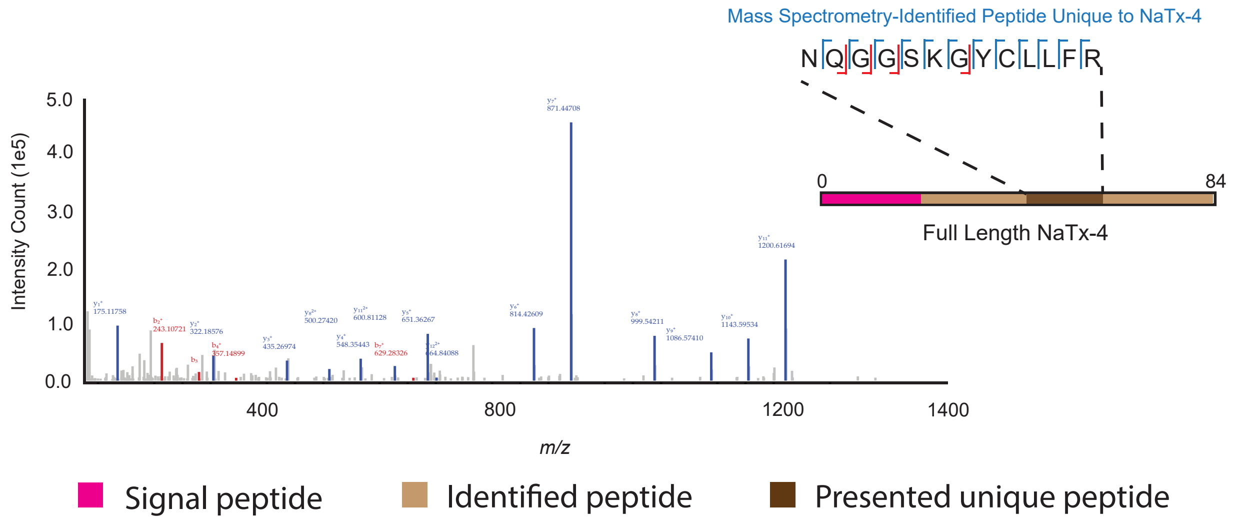

Figure S4. Summary of bottom-up proteomics data from synthetic peptide NaTx4. The tandem mass spectrum obtained via higher-energy collisional dissociation is represented with *b*-type product ions indicated in red and *y*-type ions in blue. The inset shows the sequencing of the C-terminal proteolytic peptide, which corresponds to the brown bar in the toxin sequence. The database search used the whole sequence of the toxin, but the signal peptide (indicated in magenta) was not identified, as expected. Total sequence coverage for the mature toxin = 76%, excluding the signal peptide.

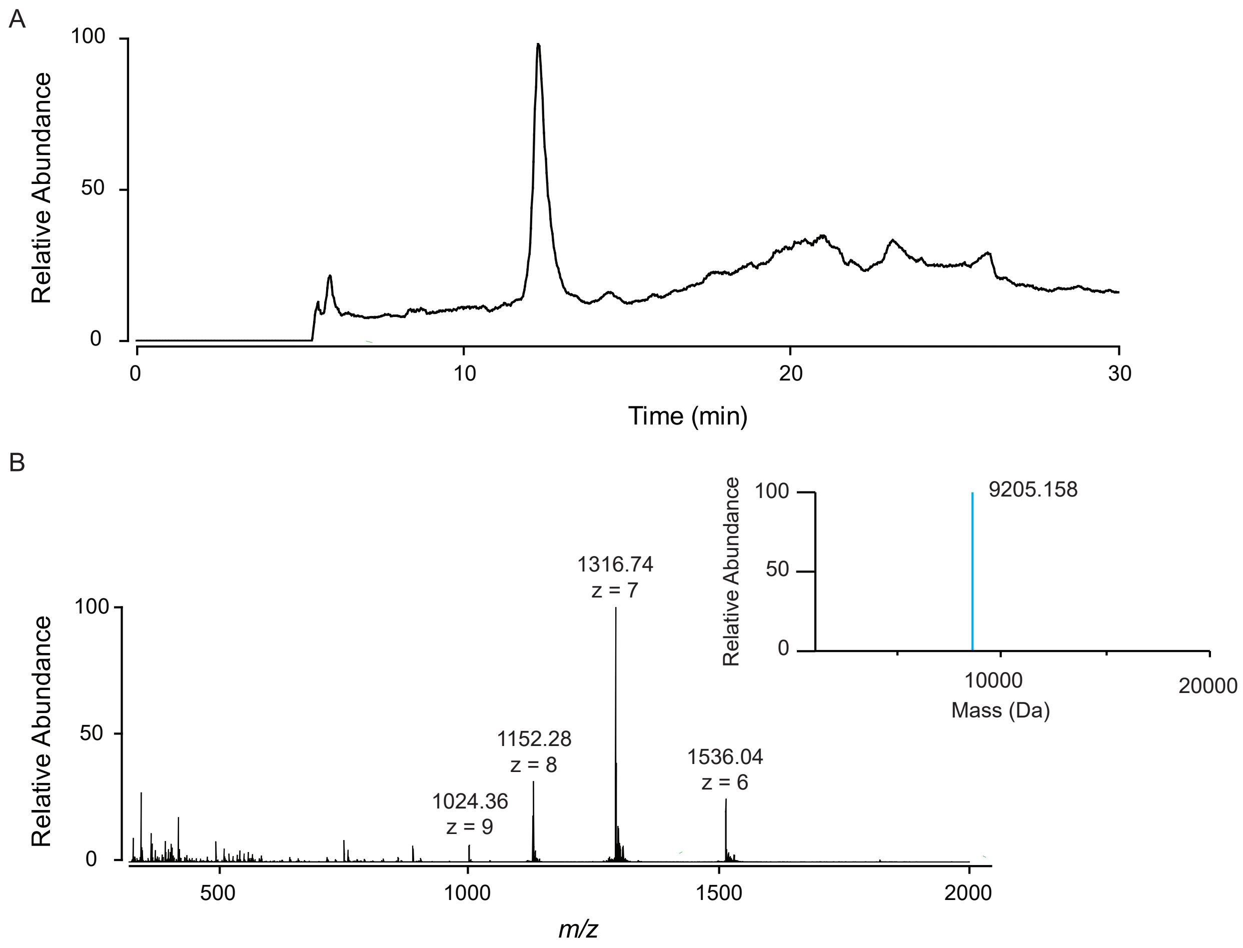

Figure S5. Intact mass analyses of synthetic NaTx13. **(A)** Chromatogram of NaTx13 during intact mass analysis. **(B)** High resolution (120,000 at 200 *m/z*) mass spectrum of the synthetic peptide toxin. Insert shows the deconvoluted mass of NaTx13; calculated mass from spectrum: 9205.158 Da; theoretical mass from sequence: 9204.16 Da; mass error: 1 Da.

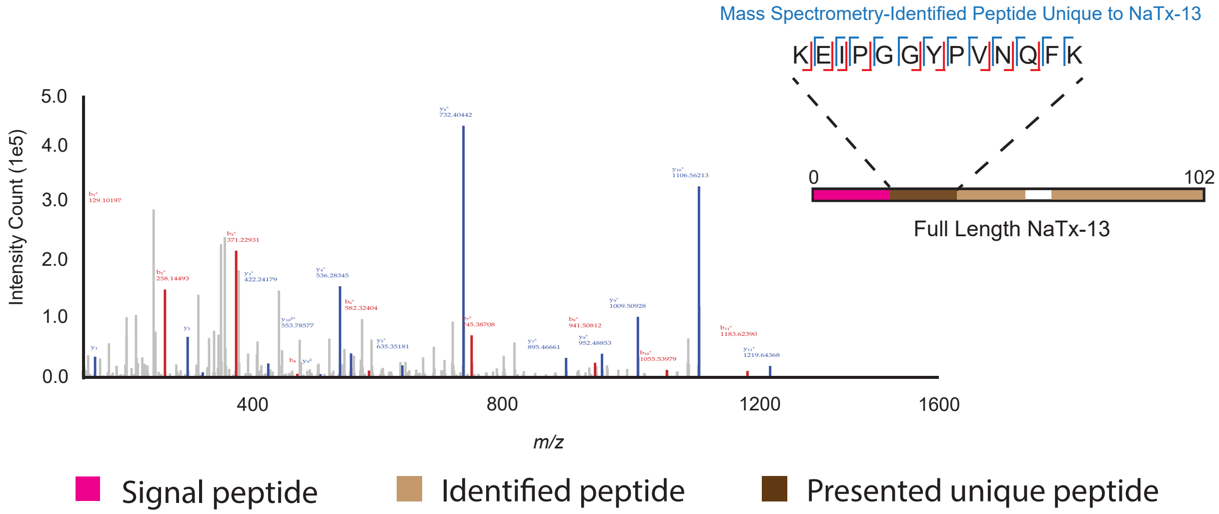

Figure S6. Summary of bottom-up proteomics data from synthetic peptide NaTx13. The tandem mass spectrum obtained via higher-energy collisional dissociation is represented with *b*-type product ions indicated in red and *y*-type ions in blue. The inset shows the sequencing of the C-terminal proteolytic peptide, which corresponds to the brown bar in the toxin sequence. The database search used the whole sequence of the toxin, but the signal peptide (indicated in magenta) was not identified, as expected. Total sequence coverage for the mature toxin = 72%, excluding the signal peptide.

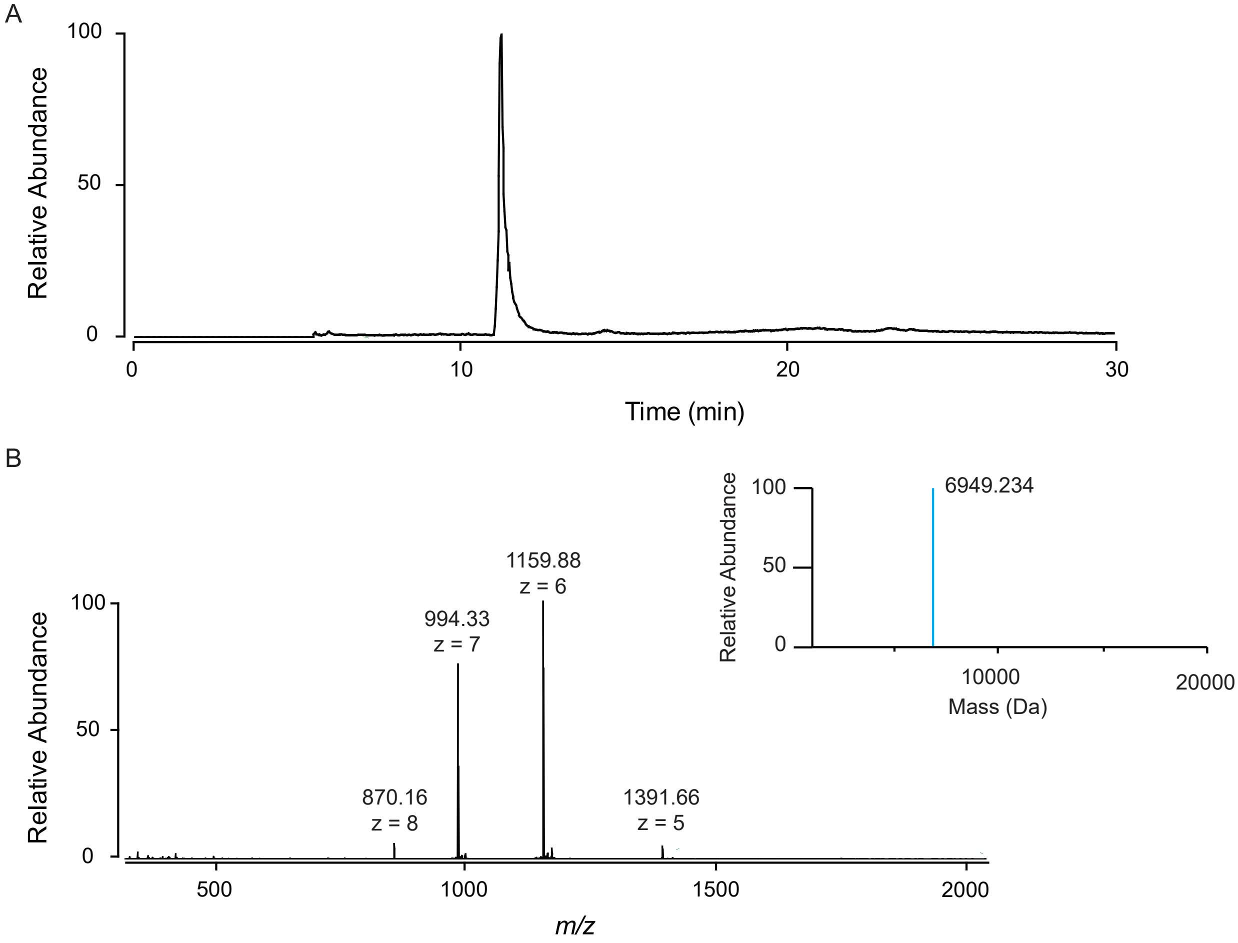

Figure S7. Intact mass analyses of synthetic NaTx22. **(A)** Chromatogram of NaTx22 during intact mass analysis. **(B)** High resolution (120,000 at 200 *m/z*) mass spectrum of the synthetic toxin protein. Insert shows the deconvoluted mass of NaTx22; calculated mass from spectrum: 6949.234 Da; theoretical mass from sequence: 6949.23 Da; mass error: 0.004 Da (0.7 ppm).

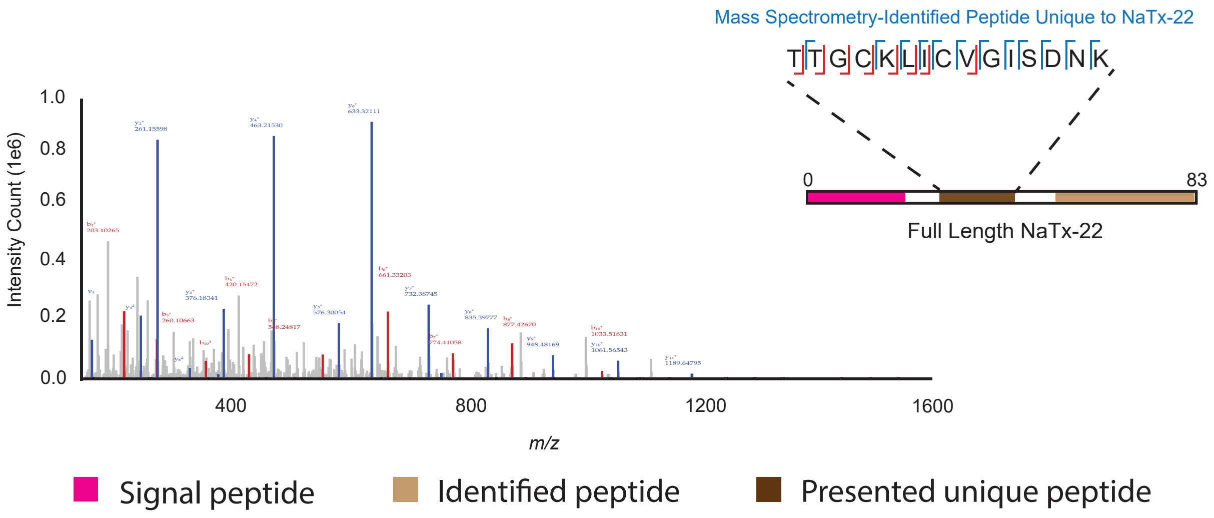

Figure S8. Summary of bottom-up proteomics data from synthetic peptide NaTx22. The tandem mass spectrum obtained via higher-energy collisional dissociation is represented with *b*-type product ions indicated in red and *y*-type ions in blue. The inset shows the sequencing of the C-terminal proteolytic peptide, which corresponds to the brown bar in the toxin sequence. The database search used the whole sequence of the toxin, but the signal peptide (indicated in magenta) was not identified, as expected. Total sequence coverage for the mature toxin = 60%, excluding the signal peptide.

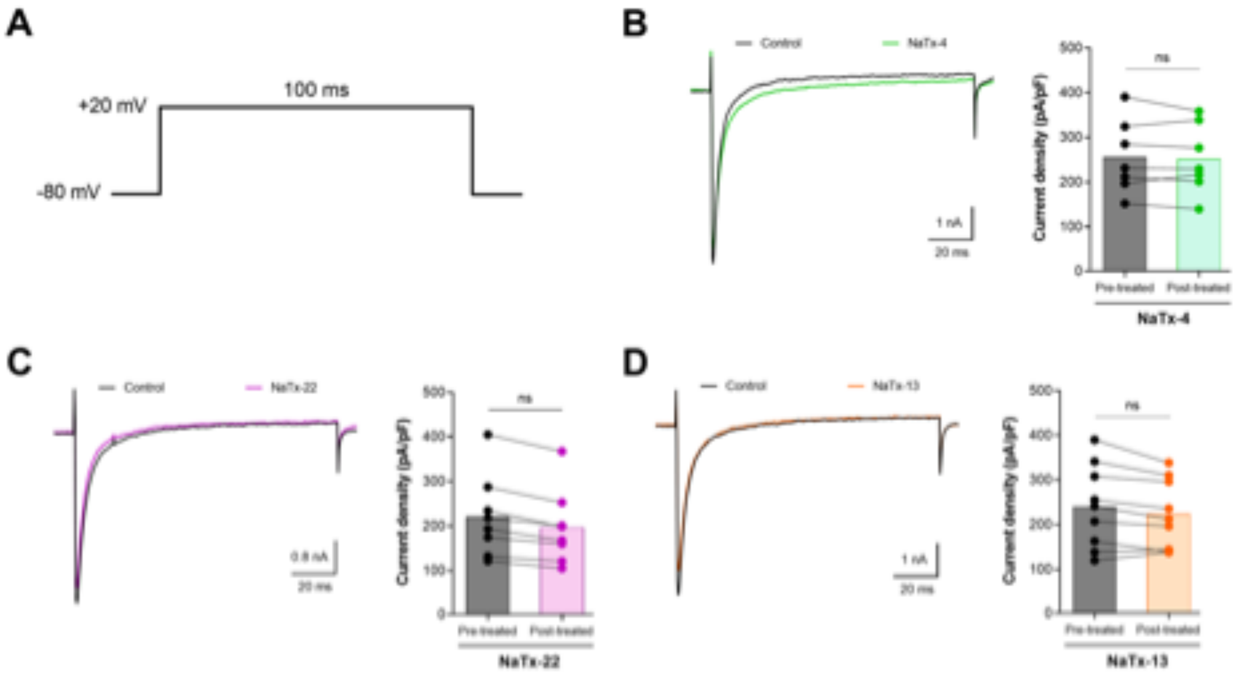

Figure S9. Effects of chemically synthesized bark scorpion peptide toxins on grasshopper mouse recombinant $OtNa_V1.8$ Na^+ current density. **(A)** Na^+ currents were elicited by a 100-millisecond depolarization to +20 mV from a holding potential of -80 mV before and after application of peptide toxins. Tetrodotoxin (TTX, 500 nM) was added to the bath solution to block endogenous TTX sensitive Na^+ currents. Representative Na^+ current traces and current density (pA/pF) recorded from whole-cell voltage-clamped ND7/23 cells transfected with $OtNa_V1.8$. Na^+ currents were recorded before and after application of peptide toxins (all toxins tested at 10 μg/mL). **(B – D)** NaTx-4 (green, n = 7 cells), NaTx-22 (purple, n = 8 cells), NaTx-13 (orange, n = 9 cells) and had no effect on $OtNa_V1.8$ activity. Filled circles represent current density values for individually recorded cells. Histograms represent mean current density reported as picoamps divided by picofarads (pA/pF, * $p < 0.05$).

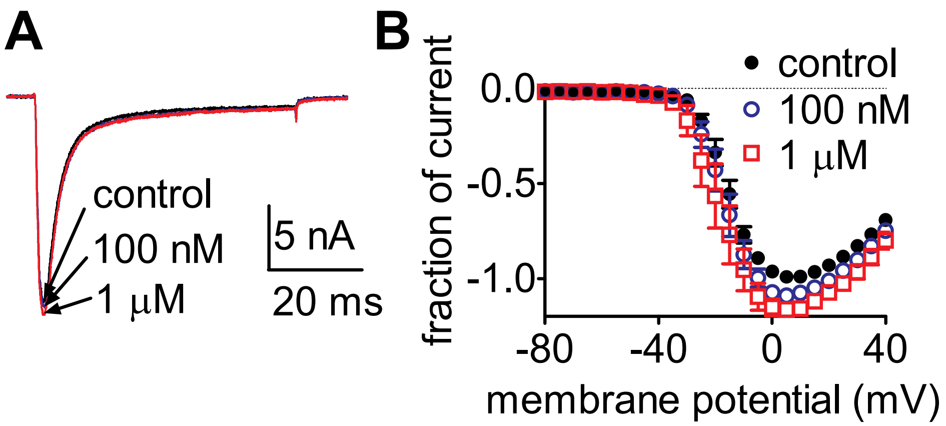

Figure S10. Effects of NaTx36 on human recombinant $hNa_V1.8$ Na^+ current density. NaTx36 did not affect hNav1.8 expressed in ND7/23 cells. **(A)** Sodium currents before and after application of NaTx36 (100 nM or 1 μM) were elicited by a 50-ms depolarizing pulse to +10 mV. **(B)** Effect of NaTx36 on the current-voltage relationship. Sodium currents were induced by 50-ms depolarizing steps to various potentials ranging from -80 to +40 mV in 5-mV increments. In **(A)** and **(B)**, cells were held at -80 mV. Each data point (mean ± S.E.) represents five separate experimental cells.

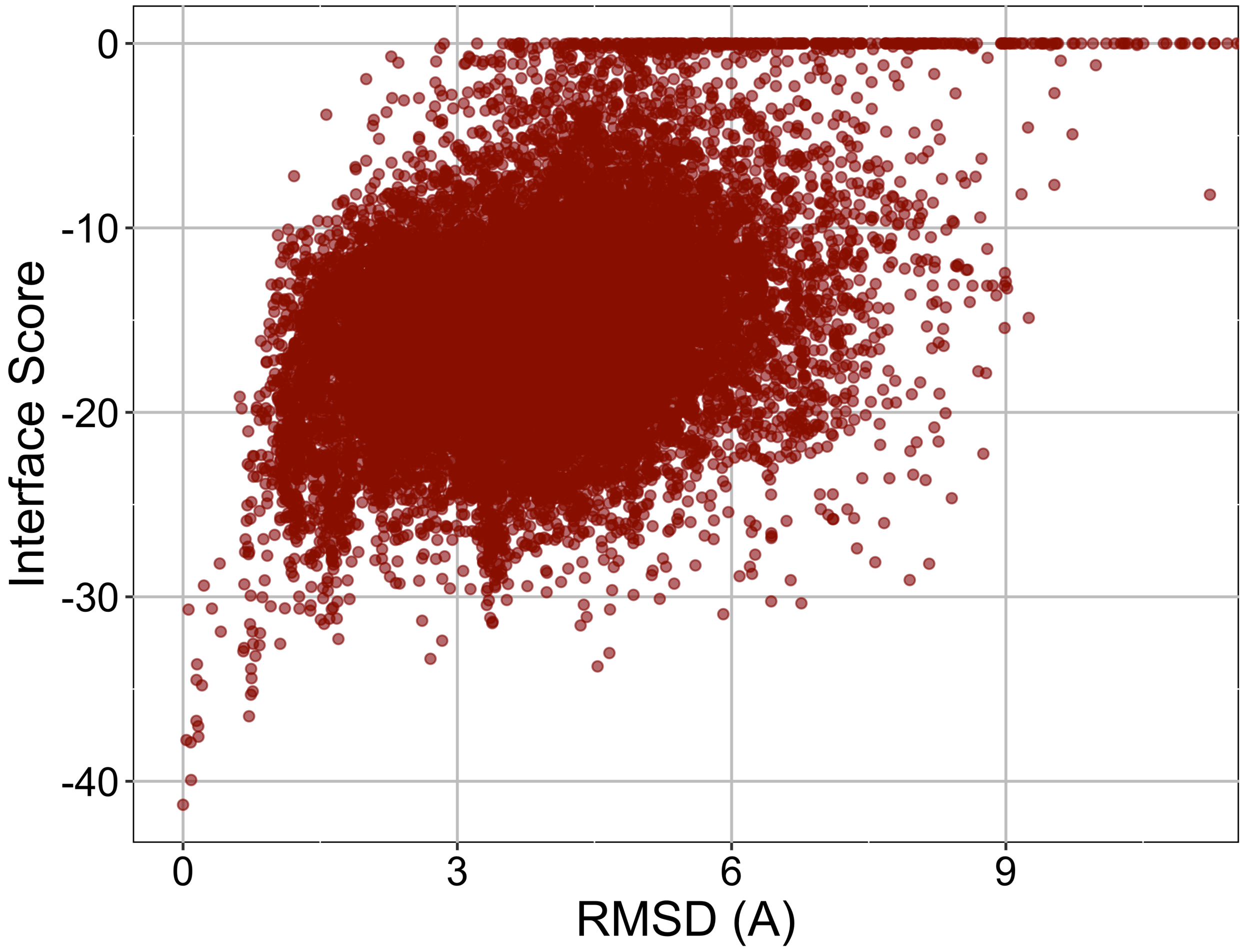

Figure S11. Interface energy minimum score representing binding conformation between NaTx36 and OtNaV1.8. Approximately 20,000 models were generated at which structures converged toward an interface energy minimum suggesting they had reached a near-native binding conformation between NaTx36 and OtNaV1.8.

OtNaV1.8			NaTx-36			
Residue Number	Residue Name	Chain	Residue Number	Residue Name	Chain	Raw Score
150	ASN	A	18	ASP	B	-475.438
815	CYS	A	19	ASN	B	-226.058
817	LYS	A	19	ASN	B	-165.762
210	GLY	A	39	TRP	B	-89.462
815	CYS	A	17	PHE	B	-84.855
150	ASN	A	17	PHE	B	-7.603
150	ASN	A	16	LEU	B	-5.895
209	ARG	A	63	ALA	B	-5.807
209	ARG	A	39	TRP	B	-4.155
209	ARG	A	40	LYS	B	-3.808
154	GLU	A	14	ALA	B	-2.954
149	GLN	A	16	LEU	B	-2.482
816	ARG	A	19	ASN	B	-2.242
807	GLN	A	38	PHE	B	-1.97
215	ARG	A	16	LEU	B	-1.042
209	ARG	A	41	GLN	B	-0.896
811	GLU	A	17	PHE	B	-0.852
806	LYS	A	17	PHE	B	-0.483
151	ASP	A	18	ASP	B	-0.255
811	GLU	A	39	TRP	B	-0.109
807	GLN	A	17	PHE	B	0.135
817	LYS	A	20	ASP	B	0.33
215	ARG	A	41	GLN	B	0.496
816	ARG	A	37	TYR	B	0.886
155	LYS	A	9	ASP	B	0.932
818	ASP	A	35	TYR	B	1.079
207	ASP	A	62	ARG	B	2.911
816	ARG	A	35	TYR	B	2.993

Table S1. Protein Interaction Z Score Assessment (PIZSA) analysis of the binding interface between OtNav1.8 and NaTx36 in our final model. Raw score: -1075.33; Z-score: 2.185.
